# A FFLUX Water Model: Flexible, Polarizable and with a Multipolar Description of Electrostatics

**DOI:** 10.1002/jcc.26111

**Published:** 2019-11-20

**Authors:** Zak E. Hughes, Emmanuel Ren, Joseph C. R. Thacker, Benjamin C. B. Symons, Arnaldo F. Silva, Paul L. A. Popelier

**Affiliations:** ^1^ Manchester Institute of Biotechnology, The University of Manchester Manchester M1 7DN United Kingdom; ^2^ Department of Chemistry The University of Manchester Manchester M13 9PL United Kingdom; ^3^ School of Chemistry and Biosciences, University of Bradford Bradford BD7 1DP United Kingdom

**Keywords:** molecular dynamics, machine learning, quantum chemical topology, water simulation, multipole moments

## Abstract

Key to progress in molecular simulation is the development of advanced models that go beyond the limitations of traditional force fields that employ a fixed, point charge‐based description of electrostatics. Taking water as an example system, the FFLUX framework is shown capable of producing models that are flexible, polarizable and have a multipolar description of the electrostatics. The kriging machine‐learning methods used in FFLUX are able to reproduce the intramolecular potential energy surface and multipole moments of a single water molecule with chemical accuracy using as few as 50 training configurations. Molecular dynamics simulations of water clusters (25–216 molecules) using the new FFLUX model reveal that incorporating charge‐quadrupole, dipole–dipole, and quadrupole–charge interactions into the description of the electrostatics results in significant changes to the intermolecular structuring of the water molecules. © 2019 The Authors. *Journal of Computational Chemistry* published by Wiley Periodicals, Inc.

## Graphical abstract

Traditional molecular force fields are inevitably limited in their accuracy and transferability. FFLUX is a new approach to force field development that aims to leverage quantum chemical topology (QCT) and machine learning to provide a quantum mechanical description the potential energy surface of systems into a force field. Here, a variety of FFLUX force fields have been created for water and their performance analyzed both at the single molecule level and for the simulation of water clusters.

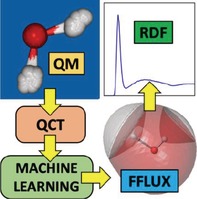

## Introduction

The increasing power of computers has enabled classical molecular dynamics (MD) simulations to tackle scientific problems of increasing complexity. Yet the ability to access increasingly large time and length‐scales has shown that the traditional (bio)molecular force fields (FFs) that are used to model the potential energy surface (PES) of the systems simulated suffer from a number of limitations.^1^ One alternative to classical MD is *ab initio* MD (AIMD) where the PES is modeled by a quantum mechanical description. However, AIMD also suffers from a number of limitations, in terms of both the computational cost and the description of the PES that it supplies, for example, traditional generalized gradient approximation (GGA) density functionals theory (DFT) functionals do not describe the medium‐to long‐range dispersion interactions between species accurately.^2^, ^3^, ^4^ As such, over the last decade, there has been significant research based around the development of a new generation of FFs for classical MD simulations.^5^, ^6^ The methods that these next‐generation FFs use, in order to provide a more accurate representation of the PES than traditional FFs, vary.^6^ However, one key issue many attempt to address is to provide a more realistic description of electrostatics than the point‐charge model of traditional FFs.

FFLUX^7^, ^8^ is a next‐generation FF that describes the interaction of atoms via the quantum chemical topology (QCT) approach.^9^, ^10^ QCT defines finite‐volume, space‐filling atoms that naturally emerge in the electron density as the so‐called basins with nuclear attractors, using the language of dynamical systems. Previous simulations^11^, ^12^, ^13^, ^14^, ^15^, ^16^ have used a QCT description to determine the high‐rank multipolar^17^, ^18^ electrostatic interactions between molecules, but the intramolecular geometry of the molecules was constrained, leading to the model not being polarizable. In the current work, we do include polarization within this context of multipolar electrostatics of (quantum) topological atoms.^19^ For that purpose, we need a more complete description of the energies involved, other than electrostatics, which is provided by a QCT method called interacting quantum atoms (IQA).^20^ Inspired by early work^21^ of our group, IQA defines intra‐atomic energy (including kinetic, electrostatic and exchange (−correlation) energy), as well as interatomic exchange‐(correlation) energy. The IQA energy contributions mentioned previously describe how energy varies, within a flexible monomer, at the atomistic level. Thanks to a recent advance,^22^ the density functional B3LYP can be used in the context of IQA, which is relevant given the level of theory used here (*vide infra*). By combining a QCT description of the PES with the machine‐learning method *kriging* (also known as Gaussian process regression), the geometry optimization and MD simulations of flexible molecules, such as peptide‐capped glycine,^23^ are made computationally tractable. FFLUX has recently been shown to describe the PES of individual molecules, thereby accurately identifying energetic minima.^24^

Water has always been a key target in the development of simulation models due to its importance in so many systems, especially biological systems. The performance of biomolecular force fields is related to the ability of accurately describing water–water and biomolecule–water interactions.^25^, ^26^, ^27^, ^28^ The complex, often atypical, nature of water^29^ has meant that developing a transferable model that can accurately describe its properties across different systems and conditions is highly challenging.^30^ At the same time, the number of water molecules in a typical biomolecular simulation means that computational cost is still a major factor in any choice of water model; hence, the enduring popularity of fixed point‐charge models with pairwise potentials, such as the SPC^31^ and TIP*n*P^32^ families. However, while moving beyond a point‐charge description of electrostatics incurs an increased computational cost, it is key to improving the accuracy and transferability of water models. A wide range of techniques are currently being developed to provide a more accurate description of the electrostatic interactions (e.g., SWM4‐(N)DP,^33^, ^34^ BK3,^35^, ^36^ MB‐pol,^37^, ^38^, ^39^ APIMD‐QDO,^40^, ^41^ AMOEBA,^42^, ^43^, ^44^, ^45^ OPC,^46^ and others^47^, ^48^, ^49^, ^50^). For a fuller discussion of these different models, each with their own advantages and disadvantages, we refer readers to two recent review articles.^30^, ^51^

The present study first describes the construction of a set of FFLUX models that describe the water monomer, determining which properties are most crucial in affecting model performance and how many configurations are required in the training ensemble. Next, the FFLUX models are applied to the simulation of water clusters, illustrating for the first time a MD simulation using FFLUX of multiple flexible, polarizable molecules at finite temperature.

## Methods

### FFLUX background

**Figure 1:**
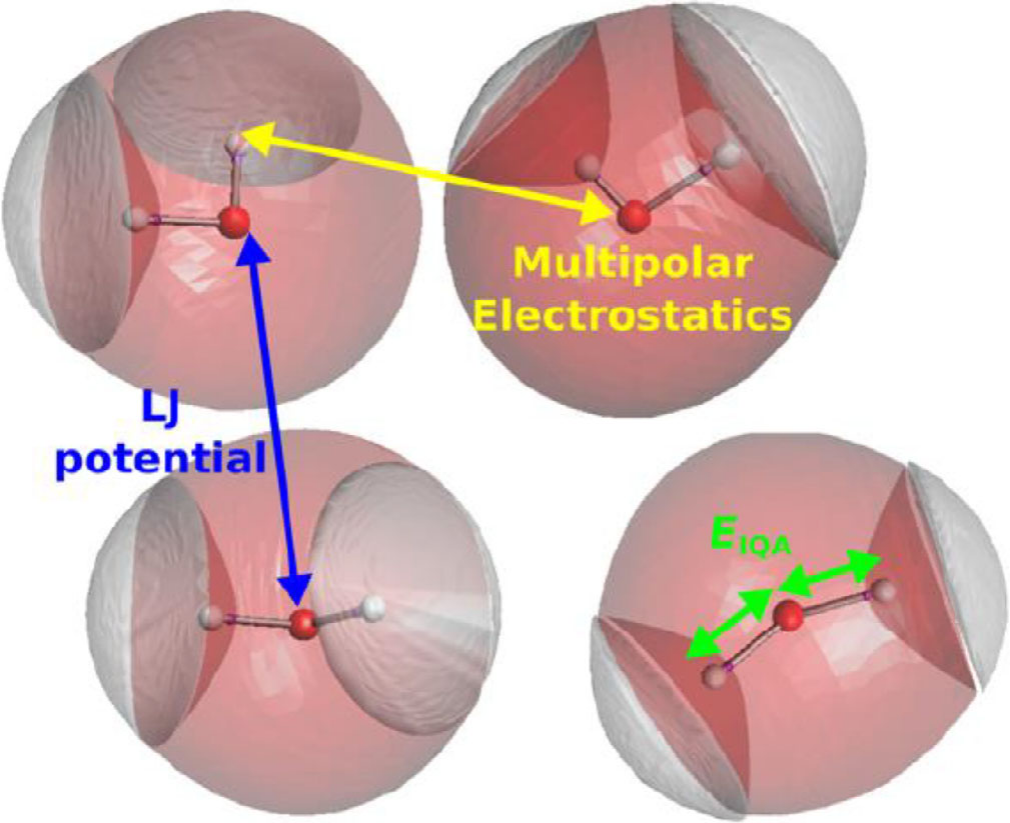
Schematic representation of the interactions that are present between atoms in the FFLUX force field. The intramolecular degrees of freedom are determined via kriging models of the atomic $E_{\mathrm{IQA}}^{A}$ energies. The electrostatic interactions between all intermolecular pairs of atoms are obtained from multipole moments predicted by kriging models. All intermolecular dispersion interactions are described by Lennard‐Jones (LJ) potentials. [Color figure can be viewed at http://wileyonlinelibrary.com]

An illustrative representation of the interaction of atoms in FFLUX simulations is shown in Figure 1. The intra‐molecular PES is described by a kriged potential based on the sum of the atomic energy terms, $E_{\mathrm{IQA}}^{A}$, one term for each of the three atoms present in each water molecule. The intermolecular interactions between pairs of atoms in different water molecules consists of an electrostatic interaction (including multipolar electrostatics) and a dispersion interaction complemented with a Pauli repulsion term (here described using a Lennard‐Jones [LJ] potential). The electrostatic interactions are described by evaluating the interaction of the atomic multipole moments. The moments are situated on the nuclear site and are also predicted by kriging models. As the configuration of a water molecule changes, the predicted multipole moments on the atoms in the water molecule will also change, thereby allowing the simulation of a polarizable water model with a multipolar description of the electrostatics. Thus, in the present FFLUX description, the flexibility of the molecule and its polarizability are directly connected. However, it would be possible to use kriging models to reproduce polarizations based on charge‐only models.^52^

In FFLUX, atomic multipole moments are described as spherical harmonics of rank *l*, where *l* = 0 corresponds to the atomic charge, *l* = 1 corresponds to the dipole (consisting of three moments), *l* = 2 corresponds to the quadrupole (five moments), and so on. This formalism is more compact than the Cartesian one, which introduces redundancies. The overall level of electrostatic interaction is then defined by evaluating all the interactions up to rank *L*, given by

(1) $$L=l_{A}+l_{B}+1$$

where *l*_*A*_ and *l*_*B*_ represent the rank of the multipole moments on atoms *A* and *B*, respectively. Thus, if *L* = 3, then the electrostatic interaction between atoms includes the monopole–monopole, monopole–dipole, monopole–quadrupole, dipole–dipole, dipole–monopole, and quadrupole–monopole interactions. In the present proof‐of‐concept study, we have limited simulations to the level of *L* = 3 and below, in order to reduce the computational cost but simulations at higher *L* are possible and will be an area of investigation in future work. Unlike the intramolecular or electrostatic interactions, the dispersion interactions between pairs of atoms are not based on kriging models but rather described by LJ potentials. Because we ultimately intend to replace these LJ potentials by kriging models, the parameters for the O and H atoms are taken (slightly modified) from a previous study^15^ and have not been optimized to any significant degree.

### Construction of FFLUX models

The development of FFLUX models for a molecule involves of a series of distinct steps:

1. Generation of an ensemble of molecular configurations.
2. Calculation of the wave function of each configuration.
3. Calculation of the atomic properties of each configuration.
4. Mapping of the atomic properties to geometric features via the kriging machine‐learning method.

We describe the computational details associated with each below (further details of the process for constructing FFLUX models can also be found in previous works^7^, ^8^, ^23^, ^24^, ^53^).

#### *Generation of configurational ensembles*

The configurational ensemble was generated based on distortion according to the normal modes of molecules via the in‐house code EROS, a method previously shown to generate an ensemble that provides a good description of the PES of a water molecule.^53^ The EROS sampling method involves taking a “seed” configuration of a molecule and stochastically sampling along the normal modes, with the constraint that both bond lengths and angle are distorted by no more than 20% from their value in the seed geometry. In the present study, the seed geometry used was the B3LYP^54^/aug‐cc‐pVTZ^55^ minimum energy configuration of a single water molecule in the gas phase with no solvent corrections applied. The same set of configurations was used for constructing both the atomic energy and atomic multipole models.

#### *DFT calculations of wave functions*

After generation of the ensemble, the wave functions of all the configurations in the ensemble were obtained from DFT calculations using the B3LYP functional and the aug‐cc‐pVTZ basis set with 6d orbitals (six components Cartesian functions) included and with no solvent corrections applied. All DFT calculations were performed using GAUSSIAN09.^56^

#### *Atomic property calculations*

In the present study, there are two sets of atomic properties of interest: the $E_{\mathrm{IQA}}^{A}$ energy of each atom and the multipole moments of each atom. These properties have been calculated using the QCT methodology, a parameter‐free approach to partitioning the wave function using only the gradient vector of the electron density.

According to the IQA description the energy of a molecule is partitioned into the sum of atomic energies, $E_{\mathrm{IQA}}^{A}$, which are composed of intra‐atomic, $E_{\text{intra}}^{A}$ (for atoms *A*), and inter‐atomic components, EinterAA', where *A'* is any atom but atom *A*.

(2) $$E_{\mathrm{IQA}}^{A}=E_{\text{intra}}^{A}+E_{\text{inter}}^{\mathrm{AA}\prime }$$

It is possible to break down the intra‐ and inter‐atomic energies further into kinetic, exchange‐correlation and electrostatic contributions and construct FFLUX models for these various different components. However, because one of our previous studies showed that a FFLUX model built at the $E_{\mathrm{IQA}}^{A}$ level performed at least as well as the combination of models built from the separate contributions,^53^ all the models in the present work are built at the $E_{\mathrm{IQA}}^{A}$ level. Note that this level is all that is needed to perform atomistic simulations. In other words, keeping track of the types of energy during a MD trajectory only serves insight, not accuracy.^53^

The multipole moments of each atom are determined via the integration of an atomic basin of the appropriate spherical tensor and weighted by the electron density, as described more fully in previous work.^18^, ^57^

The calculations of the IQA energies and multipole moments from the wave functions were performed using the program AIMAll (version 17),^58^ with the default parameter options and with the original implementation for the calculation of the two‐electron integrals (i.e., not using the “TWOe implementation”).

#### *Construction of kriging models*

Before building the kriging models of the $E_{\mathrm{IQA}}^{A}$ and atomic multipole moments, any configurations were removed from the ensemble of sample configurations where the net molecular charge is >0.001 *e* and/or the atomic integration error of AIMAll, *L*(Ω), was above a threshold value for one or more atoms. Models were constructed using *L*(Ω) = 0.0001 and 0.00005 Ha. Next, a number, denoted *N*_trn_, of the remaining configurations in the sample were randomly selected as the training set used to build the kriging model, with (some of) the remaining configurations in the sample used as a validation set, allowing the quality of kriging models to be tested.

Full methodological details of how a property of an atom can be linked to the geometrical features through kriging machine learning are given in previous work.^23^ In the present work, the kriging machine learning generates a model that expresses the atomic energies, $E_{\mathrm{IQA}}^{A}$, (and thus a molecular potential) or atomic multipole moments as a function of positions of all the atoms in the molecule. Each atomic property, *Y*, is given by

(3) $$Y=\mu^{A}+\sum_{j=1}^{N_{\mathrm{trn}}}a_{j}^{A}\exp\left[-\sum_{h=1}^{N_{\text{feat}}}\theta_{h}^{A}{\left|f_{h,j}^{A}-f_{h}^{A}\right|}^{p_{h}^{A}}\right]$$

where *μ*^*A*^ is the estimated mean value (trend model) of all the training data points, $a_{j}^{A}$ is the kriging weight of training point *j*, $\theta_{h}^{A}$ is the activity of the feature‐space described by the summation index *h*, $f_{h,j}^{A}$ is the known feature value from training point *j*, $f_{h}^{A}$ is the current feature for which prediction must be made, and $p_{h}^{A}$ relates to the smoothness of the feature space. Kriging models can be constructed by optimizing $\theta_{h}^{A}$ and/or $p_{h}^{A}$. Previous work on organic molecules has indicated that for molecules of 6–14 atoms in size optimizing $p_{h}^{A}$ as well as $\theta_{h}^{A}$ results in only limited improvement, if any, of the resulting kriging models, and comes with an increased computational cost,^24^ both in the construction of the kriging model and in its evaluation. However, to determine whether the limited effect of the optimization of $p_{h}^{A}$ holds true for water, a system with only three intramolecular degrees of freedom, kriging models were constructed with both $\theta_{h}^{A}$ and $p_{h}^{A}$ optimized and with $\theta_{h}^{A}$ optimized and $p_{h}^{A}$ fixed at 2.

The kriging models were calculated using the in‐house developed program FEREBUS,^59^ with the values of the kriging parameters optimized using particle swarm optimization in order to maximize the concentrated log‐likelihood.

### MD simulation methodology

The MD simulations were performed on clusters of water molecules. The number of water molecules in a cluster was defined as *N*_mol_, and simulations were performed with *N*_mol_ = 25, 50, 100, and 216. Furthermore, simulations were performed at electrostatic ranks *L* = 1, 2, and 3. Some simulations were performed with the water molecules held rigid in order to allow the investigation of the effects of polarizability. The Supporting Information Table S1 gives a breakdown of the different systems and the timescales each system was simulated for.

All simulations of the FFLUX water models were performed using an in‐house modified version of the DL_POLY_4.08 program.^60^ The equations of motion were integrated using the velocity Verlet algorithm with a 1 fs timestep. The temperature of the system was maintained at 300 K using the Andersen thermostat.^61^ As mentioned earlier, the intramolecular energy of each water molecule was calculated from the sum of the $E_{\mathrm{IQA}}^{A}$ kriging models. A cutoff of 10.0 Å was used for the LJ interactions with the LJ potential shifted up slightly such that the energy was equal to zero at the cutoff. The LJ parameters were taken from previous simulations of rigid QCT water models^15^ and then modified slightly to improve stability. The final parameters were *ε*_HH_ = 0.753 kJ mol^−1^; *ε*_HH_ = 0.015 kJ mol^−1^; *σ*_OO_ = 3.23 Å; *σ*_OO_ = 1.10 Å with the Lorentz–Berthelot combining rules used for the OH parameters. The multipole moments on each atom were predicted using kriging models while the electrostatic interaction between two atoms *A* and *B* was calculated using

(4) $$E_{\text{elec}}^{\mathrm{AB}}=\sum_{l_{A}l_{B}m_{A}m_{B}}Q_{l_{A}m_{A}}T_{l_{A}l_{B}m_{A}m_{B}}Q_{l_{B}m_{B}}$$

where *l* and *m*, respectively, denote the rank and component of the multipolar moment *Q*, and *T* is the interaction tensor between the multipole moments on atom *A* and atom *B*. At the current time, the electrostatic interactions are evaluated using a simple direct space approach with a cutoff of 30.0 Å (greater than the largest separation distance on any two water molecules in any of the systems) in a nonperiodic box. Work is currently ongoing to convert the code to describe electrostatic interactions via an Ewald sum approach.

In addition to the simulations of the FFLUX water systems, simulations of three alternate flexible water models were also performed in order to act as a point of comparison. The three models chosen were AMOEBA14,^43^ AMOEBA+,^44^ and MB‐pol.^37^, ^38^, ^39^ The AMOEBA14 and AMOEBA+ are different versions of water models developed with the AMOEBA FF framework using a distributed multipole analysis to go beyond a point charge description of electrostatics. The MB‐pol water model has been designed to capture many‐body terms and includes explicit terms for one‐, two‐ and three‐body interactions and with polarization described via a modified Thole‐type mode. All three models have been shown to reproduce many of the key parameters/features of water with a high degree of accuracy.

The simulation of MB‐pol water was performed using the MB‐pol plugin to OpenMM.^62^ The current implementation of MB‐pol in OpenMM does not allow cluster simulations in the NVT ensemble. As such a cluster of 216 water molecules was placed in a 60 × 60 × 60 Å^3^ periodic cell, sufficient to ensure the cluster was effectively *in vacuo* and equivalent to the setup of the FFLUX simulations. A 10.0 Å cutoff was used for the van der Waals interactions and short‐range electrostatics, with the long‐range electrostatics evaluated using an Ewald sum. The system was simulated in the NVT ensemble for 10 ps with a 0.2 fs timestep.

The simulations of the AMOEBA14 and AMOEBA+ models were performed using the TINKER program.^63^ Clusters of 216 water molecules were simulated for 1 ns. Similar to the MB‐pol model, the clusters of water molecules were simulated in the NVT ensemble in a box of 75 × 75 × 75 Å^3^, thereby again introducing a “drop of water in a large box.” A 10.0 Å cutoff was used for the van der Waals interactions and short‐range electrostatics, with the long‐range electrostatics evaluated using an Ewald sum. The temperature was maintained using the Andersen thermostat.

## Results and Discussion

### Performance of kriging water models

The description of a FFLUX PES is dependent on the kriging machine‐learning models that have been developed. In brief, there are a number of parameters that can be varied during the generation of kriging models, and a series of different models has been systematically constructed in order to determine the most appropriate model(s) to use in the MD simulations.

FFLUX models were constructed for a varying number of training points: *N*_trn_ = 50, 75, 125, 250, 500 and 1000; for *L*(Ω) = 0.0001 and 0.00005 Ha; and with $p_{h}^{A}$ = 2 and optimized, so in total for 24 = 6 × 2 × 2 different models. Typically, the performance of FFLUX models increases with *N*_trn_ but so does the computational cost of both construction and evaluation.^24^ Thus, before performing the MD simulations, we systematically evaluated the performance of the different models.

**Figure 2:**
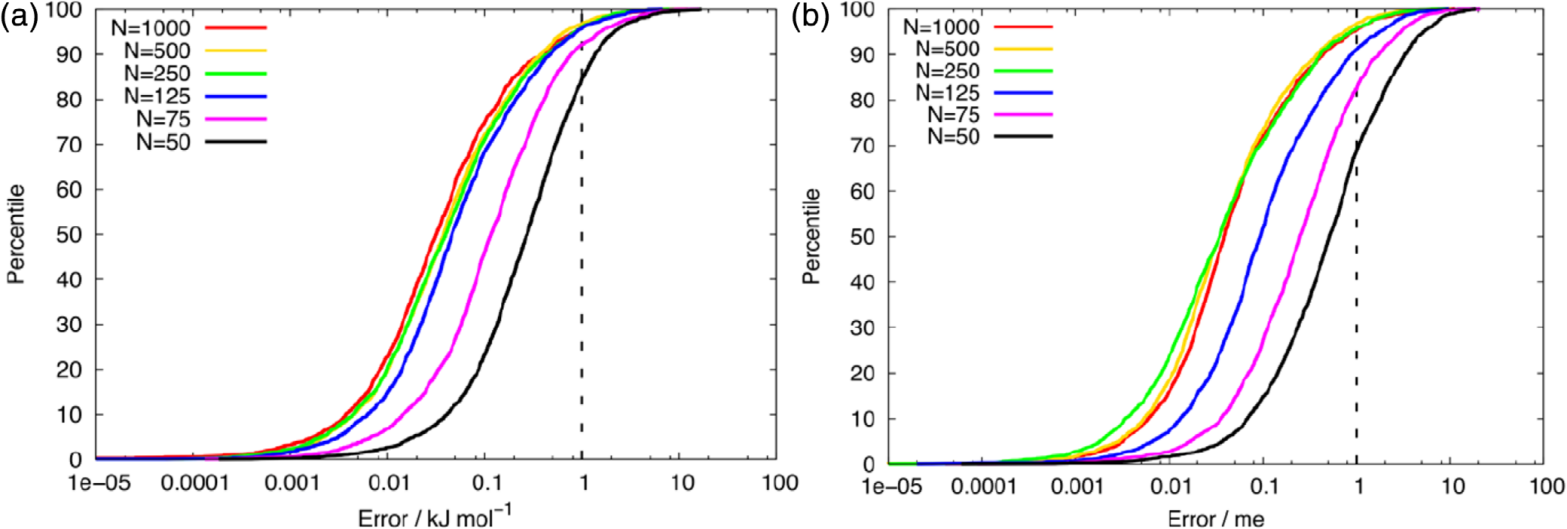
S‐curves showing the prediction error of the sum of (a) the atomic energies and (b) the atomic charges for models constructed with varying *N*_trn_, $p_{h}^{A}$ fixed at 2 and *L*(Ω) = 0.00005 Ha. [Color figure can be viewed at http://wileyonlinelibrary.com]

An initial test of the quality of the generated FFLUX model is its ability to reproduce the sum of the atomic properties of configurations in the validation set. The prediction error of a configuration is the absolute difference between the sum of the atomic energies/charges calculated from AIMAll and those calculated using FFLUX. A plot of prediction error (x‐axis) against the cumulative percentage (y‐axis) of configurations in the validation set results in a so‐called S‐curve. An S‐curve gives an indication of the quality of a given kriging model from the position of the curve on the x‐axis (the lower the values the better the model), its gradient (the steeper the curve the better the model) and the shortness of the tail near 100% (the shorter the better). Figure 2 shows exemplar S‐curves for models constructed with different numbers of in the training set and with $p_{h}^{A}$ fixed at 2 and *L*(Ω) = 0.00005 Ha, and Tables 1 and 2 give the mean absolute errors for the different models. In addition, Figures S1 and S2 in the Supporting Information show S‐curves for the *E*_IQA_ energies of various models, whereas Figures S3 and S4 show S‐curves for the molecular charge of various models.

**Table 1:** The MAEs in the prediction of the sum of the $E_{\mathrm{IQA}}^{A}$ atomic energies (kJ mol^−1^) for the configurations in the validation ensembles for the various models constructed.

*N*_trn_ a	*L*(Ω)^b^ = 0.0001 Ha		*L*(Ω) = 0.00005 Ha	
	$p_{h}^{A}$ = 2	$p_{h}^{A}$ Opt	$p_{h}^{A}$ = 2	$p_{h}^{A}$ Opt
50	0.552	0.534	0.620	0.561
75	0.390	0.210	0.379	0.193
125	0.210	0.114	0.201	0.126
250	0.254	0.051	0.184	0.033
500	0.209	0.026	0.165	0.022
1000	0.242	0.029	0.181	0.033

a Number of configurations in training ensemble.

b Atomic integration error cutoff.

**Table 2:** The MAEs in the prediction of the sum of the atomic charges (m*e*) for the configurations in the validation ensembles for the various models constructed.

*N*_trn_ a	*L*(Ω)^b^ = 0.0001 Ha		*L*(Ω) = 0.00005 Ha	
	$p_{h}^{A}$ = 2	$p_{h}^{A}$ Opt	$p_{h}^{A}$ = 2	$p_{h}^{A}$ Opt
50	1.71	0.75	1.27	0.57
75	0.85	0.34	0.69	0.24
125	0.39	0.18	0.36	0.17
250	0.30	0.05	0.19	0.06
500	0.19	0.03	0.16	0.02
1000	0.29	0.02	0.22	0.01

a Number of configurations in training ensemble.

b Atomic integration error cutoff.

Consistent with previous results, an increase in *N*_trn_ typically led to an increase in model performance, but with diminishing returns (Figs. 2, S1–S4, and Tables 1 and 2). For models where $p_{h}^{A}$ was fixed to 2, there was very little improvement in the performance of models with *N*_trn_ > 125 or 250 for the *E*_IQA_ or molecular charge, respectively. In fact, increasing *N*_trn_ from 500 to 1000 actually resulted in an increase of the mean absolute error (MAE). Table 2 shows that reducing *L*(Ω) from 0.0001 to 0.00005 Ha typically led to a modest improvement in the atomic charge models, which became more significant as *N*_trn_ was increased. Likewise, optimizing $p_{h}^{A}$ resulted in only a small improvement in models when *N*_trn_ ≤ 75 but a significant improvement when *N*_trn_ ≥ 250. Overall, the quality of all models was very high, with errors of less than 1 kJ mol^−1^ and 2 m*e* (milli‐electron) in the *E*_IQA_ energy and molecular charge, respectively. It is ultimately desirable to use FFLUX models that are constructed from as few training points as possible.

**Figure 3:**
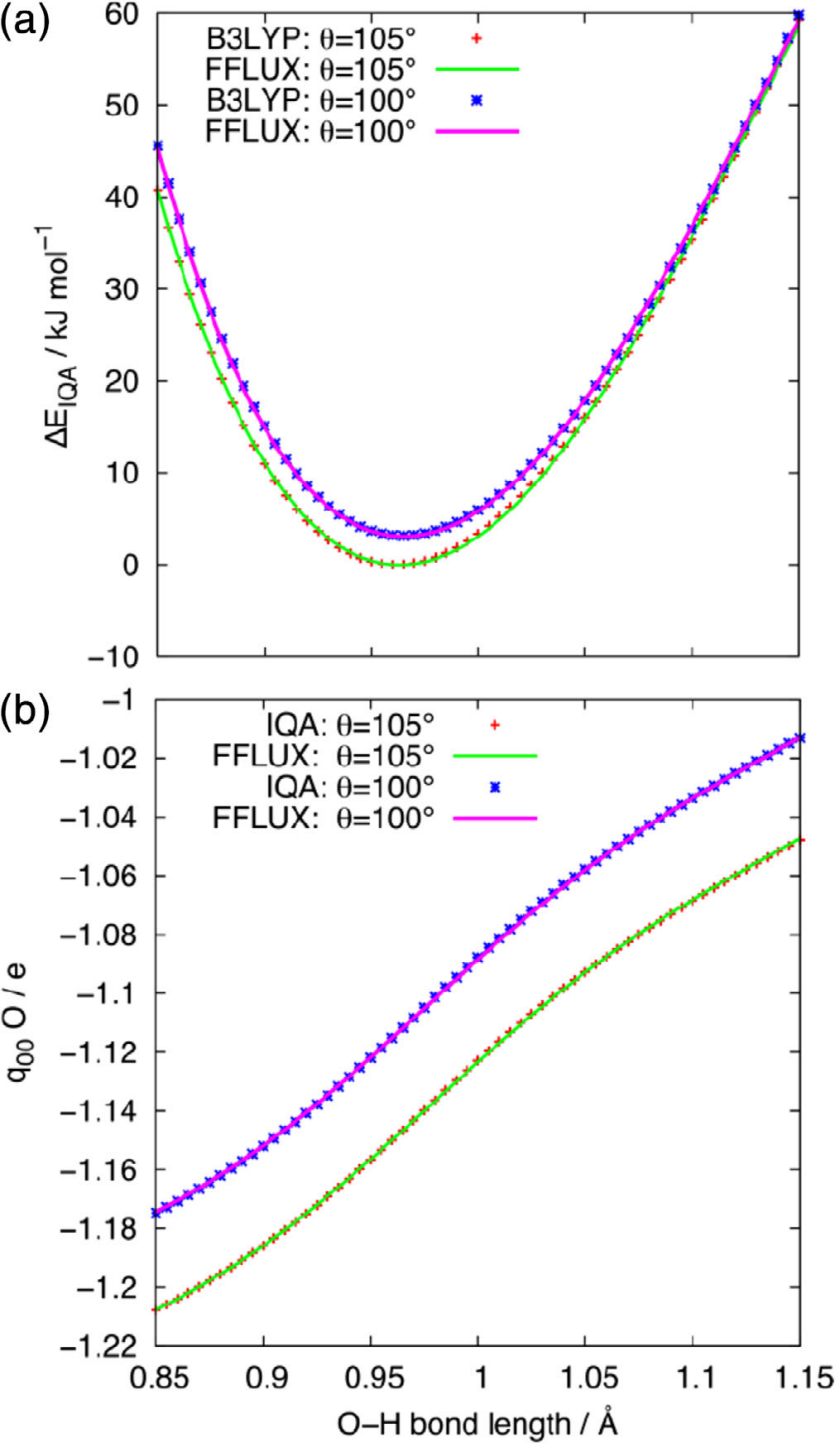
Comparison of the B3LYP/IQA and FFLUX calculations of (a) the *E*_IQA_ energy, relative to the global minimum and (b) the atomic charge on the oxygen atom for water molecular configurations with one O─H bond length varied (the other bond length is fixed at 0.9619 Å). In the legends, θ corresponds to the HOH bond angle, while B3LYP, IQA, and FFLUX correspond to the method used to perform the calculation. [Color figure can be viewed at http://wileyonlinelibrary.com]

In order to further test the *N*_trn_ = 50, *L*(Ω) = 0.00005 Ha, $p_{h}^{A}=2$ model, we investigate how well it described the PES associated with the stretching of one of the O─H bonds. One of the O─H bond lengths was increased from 0.85 to 1.15 Å in 0.005 Å steps, while the other O─H bond was fixed at 0.9619 Å, and the HOH bond angle fixed at either 105.05 or 100°. These two values were chosen for analysis as 105.05° is the bond angle of the optimized molecule and 100° is the mode bond angle of the models during the MD simulations (*vide infra*). At each point, the energy of the molecule and charge on the oxygen atom was calculated using B3LYP or IQA and compared against the values of the same properties predicted by the FFLUX models. Figure 3 shows that even for this model constructed from an ensemble containing only 50 configurations, FFLUX reproduces the molecular energy and atomic charge excellently.

From the results of the above analysis, it was decided that the *N*_trn_ = 50, *L*(Ω) = 0.00005 Ha, $p_{h}^{A}=2$ model will be used as the standard model in the MD simulations. However, a few simulations will be performed using the *N*_trn_ = 500, *L*(Ω) = 0.00005 Ha, $p_{h}^{A}=2$ model, to investigate if any significant differences in water behavior are observed for higher quality models.

### Simulations of water clusters

**Figure 4:**
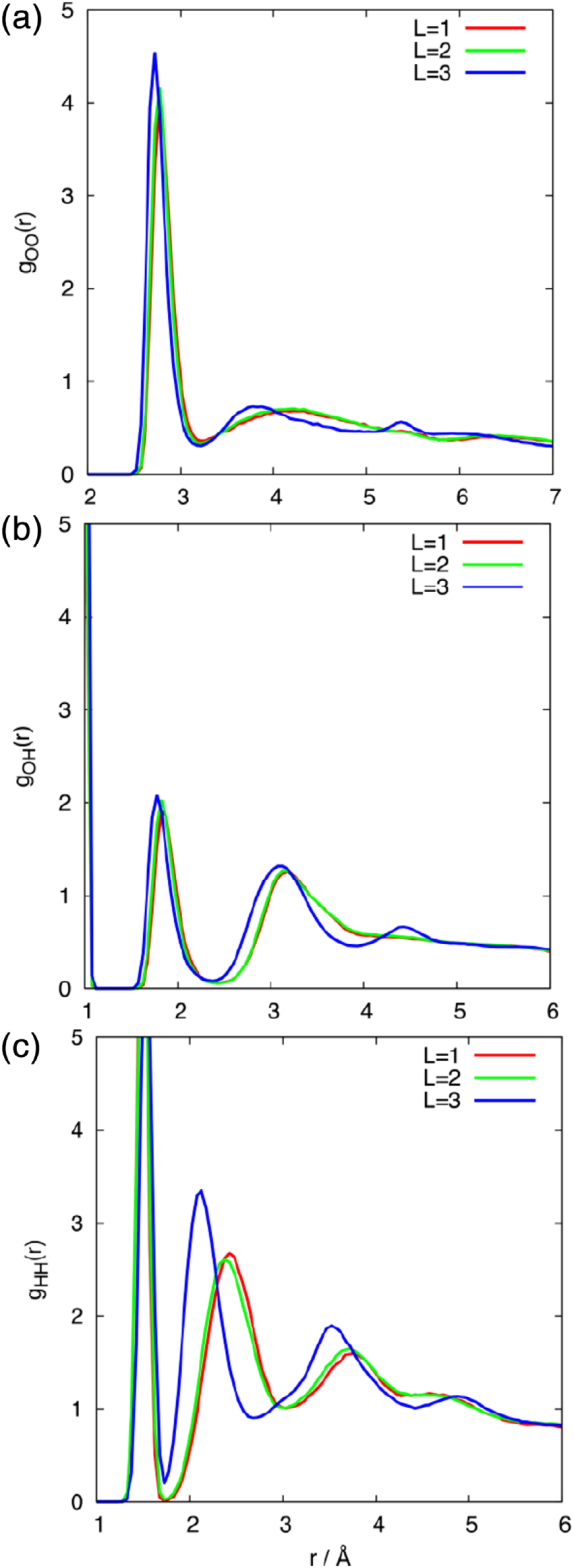
Radial distribution functions, *g*(*r*), taken from FFLUX simulations of a cluster containing 50 molecules with different levels of electrostatic interaction: (a) *g*_OO_(*r*), (b) *g*_OH_(*r*) and (c) *g*_HH_(*r*). [Color figure can be viewed at http://wileyonlinelibrary.com]

The radial distribution functions, *g*(*r*), for a water cluster of 50 molecules are shown in Figure 4 for *L* = 1, 2, and 3, for FFLUX models constructed from the *N*_trn_ = 50 data. Figures S5–S7 in the Supporting Information show that increasing *N*_trn_ to 500 does not result in significant differences to *g*(*r*), and that varying the number of water molecules in the cluster affects the height of the peaks but not their position. In spite of the lack of optimization of the LJ parameters, the position of the first peaks in *g*_OO_(*r*) is in good agreement with the experimental value of bulk water (*r* = 2.78 Å). The positions of the second peaks are lower than the experimental value for bulk water (*r* = 4.53 Å) and show more variation with the level of electrostatic description, with the *L* = 3 peak shifted to an even lower distance compared to *L* = 1 and *L* = 2. Furthermore, an extra maximum appears in the *g*_OO_(*r*) profile at ~5.35 Å for *L* = 3. For *g*_OH_(*r*) the *L* = 1 and *L* = 2 profiles are equivalent, whereas in the case of *L* = 3, the maxima are again shifted to slightly lower *r*, and an extra peak is present at longer distances (~4.50 Å). The most significant difference with *L* is observed in the *g*_HH_(*r*) profiles. While *L* = 1 and *L* = 2 show relatively minor differences, the second maximum in the *L* = 3 profile shifts from 2.43 to 2.13 Å (note the first peak corresponds to the intra‐molecular H─H distance).

**Figure 5:**
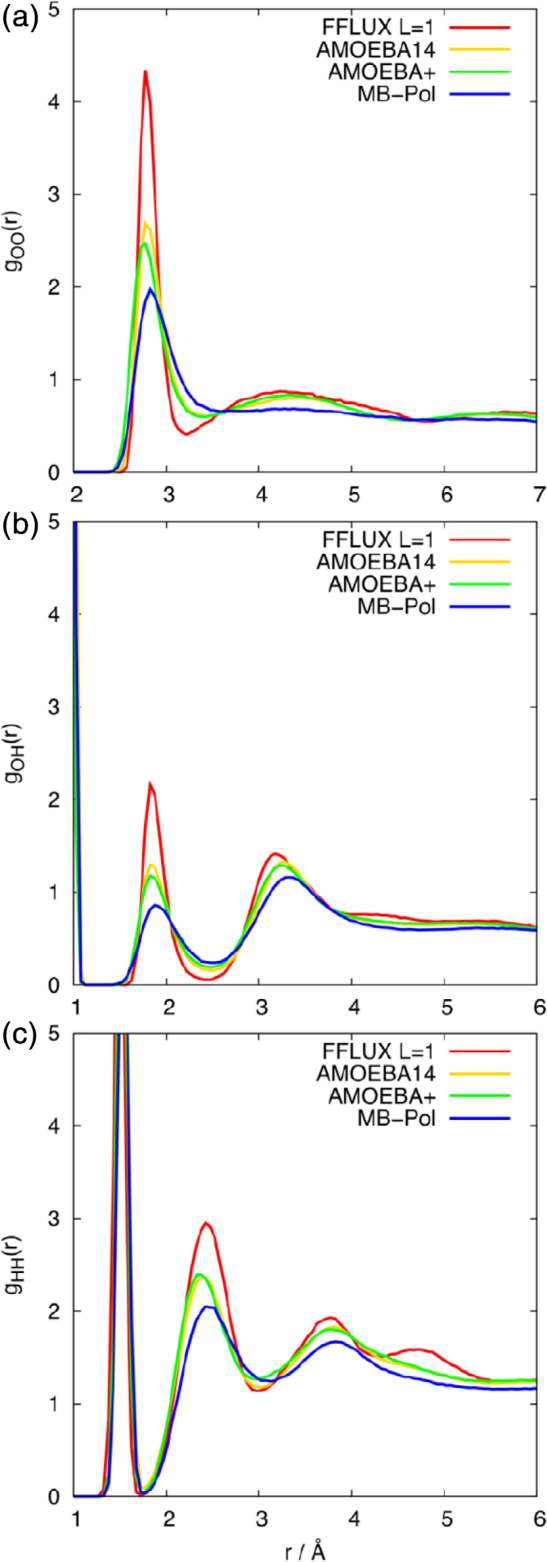
Radial distribution functions, *g*(*r*), of the FFLUX *L* = 1 model and the AMOEBA14, AMOEBA+, and MB‐pol water models: (a) *g*_OO_(*r*), (b) *g*_OH_(*r*), and (c) *g*_HH_(*r*) for a cluster of 216 molecules. [Color figure can be viewed at http://wileyonlinelibrary.com]

FFLUX has been evaluated against three other flexible, polarizable water models by comparing the *g*(*r*) for FFLUX (*L* = 1) against those of AMOEBA14, AMOEBA+ and MB‐pol (Fig. 5). All three of these models give *g*(*r*) profiles for bulk water that are in excellent agreement with experimental data. The positions of the peaks in the *g*(*r*) vary only slightly with model but there is considerable difference in the height of the peaks. The AMOEBA14 and AMOEBA+ profiles are similar, with AMOEBA+ having a slightly lower peak height. MB‐pol not only has a lower peak height than the AMOEBA models have but also has no minimum in the *g*_OO_(*r*) profile. It should be noted that the differences between *g*(*r*) of bulk water and a cluster of water molecules are greater in the case of MB‐pol than for the AMOEBA (and FFLUX) models (Supporting Information Fig. S8). In the simulation of bulk water, the MB‐pol model does have a minimum in *g*_OO_(*r*) at ~3.4 Å, suggesting that the MB‐pol model is more sensitive to differences in the water environment than the other models. FFLUX predicts peak heights significantly larger than the other three models do, which suggests that the resulting water cluster is overstructured. The intermolecular structuring of water models is determined by the balance between the electrostatic interactions (that enforce a tetrahedral geometry) and the dispersion interactions (that disrupt the H‐bonding network^2^, ^4^, ^29^, ^64^). As mentioned earlier, the parameters for the LJ potential in FFLUX were not optimized for these models (beyond ensuring that they resulted in stable clusters), and thus the dispersion interactions between molecules are probably too weak in comparison with the electrostatic interactions. This is an area for future development in FFLUX. An alternative explanation for overstructuring of the water may be the sensitivity of atomic multipoles, especially at higher *l*, to the environment. Previous work has shown that the dipole and quadrupole moments calculated via a QCT approach quickly converge to a stable value with cluster size. However, this is another area of future investigation for FFLUX.^65^

**Figure 6:**
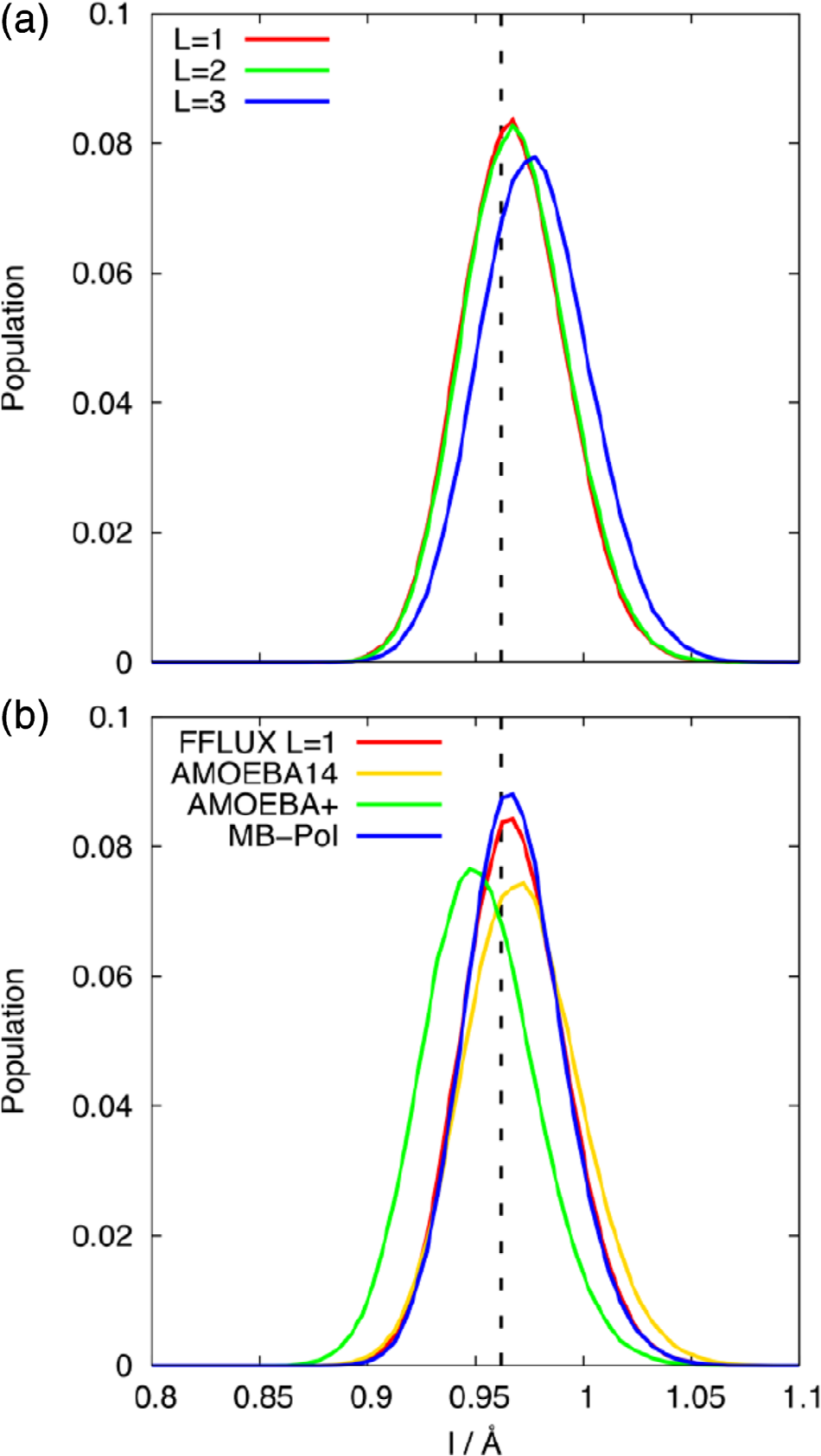
The population distributions of the O─H bond lengths from (a) simulation of a cluster containing 50 water molecules using the FFLUX models at *L* = 1, 2, and 3, and (b) simulation of a cluster of 216 water molecules with FFLUX *L* = 1, AMOEBA14, AMOEBA+ and MB‐pol water models. The dashed lines give the equilibrium bond length of a single water molecule in the gas phase, 0.9619 Å. [Color figure can be viewed at http://wileyonlinelibrary.com]

**Figure 7:**
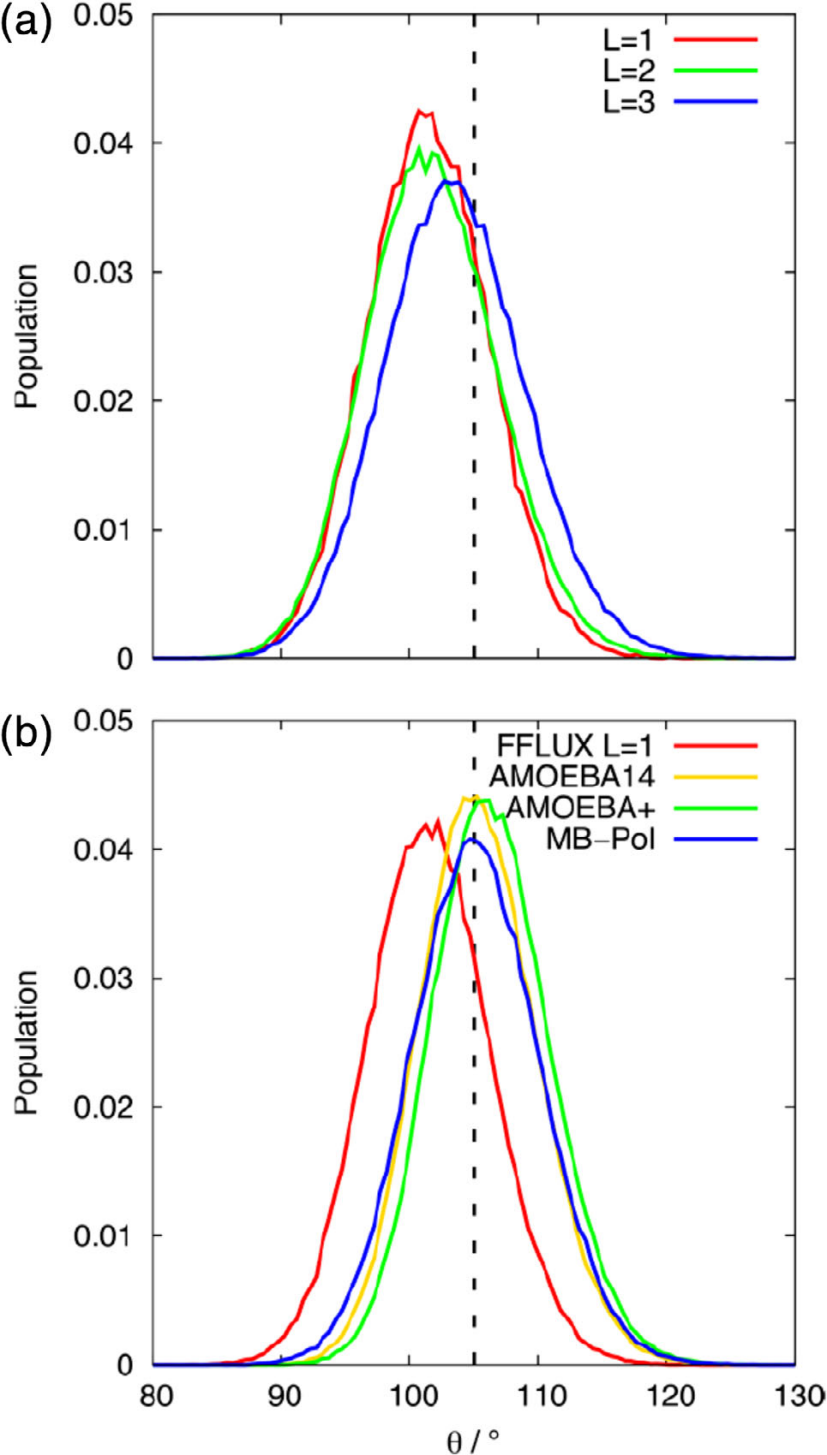
The population distributions of the HOH bond angle from (a) simulation of a cluster containing 50 water molecules using the FFLUX models at *L* = 1, 2 and 3 and (b) simulation of a cluster of 216 water molecules with FFLUX *L* = 1, AMOEBA14, AMOEBA+, and MB‐pol water models. The dashed lines give the equilibrium bond angle of a single water molecule in the gas phase, 105.05°. [Color figure can be viewed at http://wileyonlinelibrary.com]

From the above results, it is apparent that simulations with *L* = 1 and *L* = 2 show very similar *g*(*r*) profiles but that simulations with *L* = 3 results in significant changes to the intermolecular structuring of the water molecules. To determine the relationship between the intermolecular structuring and intramolecular degrees of freedom, the distribution of the bond lengths and bond angle of the water molecules was calculated. For *L* = 1 and *L* = 2 systems, the bond length distribution (Fig. 6) is centered at the B3LYP bond length of a gas molecule, 0.9619 Å, in very close agreement with the experimental gas phase O─H bond length of 0.9578 Å,^66^ and slightly lower than the experimental bond length of liquid water, 1.01 Å.^67^ When *L* = 3, the distribution is shifted to slightly greater lengths (mode bond length of 0.975 Å). The distributions of the O─H bond length of the FFLUX models are very similar (Fig. 6b) to that of the AMOEBA14 and MB‐pol models (while the AMOEBA+ model has a distribution shifted to a slightly lower length). The bond angle distributions (Fig. 7) of the FFLUX models are shifted to lower angles than the B3LYP gas phase equilibrium bond angle of 105.05° (the experimental gas phase value is 104.47°^66^). In contrast, the AMOEBA14, AMOEBA+, and MB‐pol models all give distributions centered around the gas phase bond angle of ~105°. The distribution of the FFLUX models does vary with *L*: for the *L* = 1 model the peak is at ~100°, the mode bond angle for *L* = 2 is the same as for *L* = 1, but there is a small increase in the population of bond angles that are greater than 106°. For *L* = 3 the peak in the distribution is at 103.5°, which is closer to gas phase equilibrium but still lower than the angle predicted by the other AMOEBA and MB‐pol models. The intramolecular geometry of the FFLUX water models is not significantly affected by cluster size or increasing the number of training points (see Figs. S9 and S10 in the Supporting Information).

The distributions of atomic charge on the oxygen and hydrogen atoms of the water molecules (which is a function of the intramolecular geometry of each water molecule) are shown in Figure S11 in the Supporting Information. Overall, the population distribution of the atomic charges is quite similar for *L* = 1, 2, and 3 suggesting that the difference in the intermolecular structuring is largely due to the incorporation of the charge–quadrupole, dipole–dipole, and quadrupole‐charge terms rather than resulting from differences in intramolecular geometry or atomic multipole moments. To confirm this hypothesis, we performed simulations where the internal geometry of the water molecules was held fixed (i.e., the water molecules were not polarizable). As the internal geometry of these models is fixed, the multipole moments on atoms will not differ with *L*. The *g*(*r*) profiles for these simulations (Supporting Information Figs. S12–S14) show some differences in peak heights, but the peak positions and overall shape of the profiles is similar to those of the flexible and polarizable models. As such we can be confident that it is the incorporation of the extra electrostatic terms into the *L* = 3 model that results in the different intermolecular structuring of the water molecules.

## Conclusions

Simulations of a flexible, polarizable water models with multipolar description of electrostatics have been performed by combining an IQA description of the PES of a water molecule with the machine learning method kriging. It has been found that using as few as 50 configurations for training the kriging models is sufficient to reproduce the intramolecular PES and atomic charges of a water molecule. The intermolecular structuring of the water molecules is also unaffected by increasing the number of configurations used to construct the kriging models. It is found that, while incorporating charge–dipole interactions into the description of the electrostatics results in only minor differences, the incorporation of charge–quadrupole, dipole–dipole, and quadrupole–charge interactions results in significant changes to the intermolecular structuring of the water molecules (but not to their intramolecular geometries). The positions of the peaks in the *g*(*r*) profiles indicate that FFLUX models are in reasonable agreement with existing data but that the water is somewhat overstructured, due to an imbalance between the electrostatic and dispersion interactions. Unlike the intramolecular degrees of freedom or atomic multipole moments, the dispersion interactions between molecules are, presently, simply accounted for via Lennard‐Jones potentials. In the short term, the overstructuring can be addressed by optimization of the parameters used in the Lennard‐Jones potentials and/or using an alternate potential form (such as the Buckingham potential) with optimized parameters. In the longer term, the aim is to employ machine learning potentials to also describe the intermolecular dispersion interactions.

## Supporting information


**Appendix S1**: Supporting Information
