# Supplementary material for: A FFLUX Water Model: Flexible, Polarizable and with a Multipolar Description of Electrostatics
Source: J Comput Chem. 2019 Nov 20;41(7):619–28. doi: 10.1002/jcc.26111 (PMC7004022; doi:10.1002/jcc.26111)
Supplement: Supplementary file 1 — Appendix S1: Supporting Information [file JCC-41-619-s001.pdf]

Supporting Information

A FFLUX Water Model: Flexible, Polarizable and with a Multipolar Description of Electrostatics

Zak E. Hughes,[1,2,3] *Emmanuel Ren,*[1,2] *Joseph C. R. Thacker,*[1,2] *Benjamin C.B. Symons*[1,2] *, Arnaldo F. Silva*[1,2] *and Paul L. A. Popelier*[1,2,*]

[1] Manchester Institute of Biotechnology, The University of Manchester, Manchester, M1 7DN, UK

[2] School of Chemistry, The University of Manchester, Manchester, M13 9PL, UK

[3] School of Chemistry and Biosciences, University of Bradford, Bradford, BD7 1DP, UK

*To whom correspondence should be addressed:

Phone: +44 161 3064511. E-mail: pla@manchester.ac.uk

Contents

Table S1: Summary of MD simulations performed

Figure S1: S-curves of FFLUX models for the E_{IQA} energy with p_h^A fixed

Figure S2: S-curves of FFLUX models for the E_{IQA} energy with p_h^A optimized

Figure S3: S-curves of FFLUX models for the molecular charge with p_h^A fixed

Figure S4: S-curves of FFLUX models for the molecular charge with p_h^A optimized

Figure S5: $g(r)$ profiles of water from simulations of FFLUX models with L=1

Figure S6: $g(r)$ profiles of water from simulations of FFLUX models with L=2

Figure S7: $g(r)$ profiles of water from simulations of FFLUX models with L=3

Figure S8: $g(r)$ profiles of water from simulations of bulk water with three polarizable models

Figure S9: Probability distributions of O-H bond lengths

Figure S10: Probability distributions of HOH bond angle

Figure S11: Probability distributions of atomic charges on oxygen and hydrogen atoms of FFLUX models

Figure S12: $g(r)$ profiles of water for simulations of rigid FFLUX models with L=1

Figure S13: $g(r)$ profiles of water for simulations of rigid FFLUX models with L=2

Figure S14: $g(r)$ profiles of water for simulations of rigid FFLUX models with L=3

Table S1. Summary of the different simulations performed and the timescales simulated for each.

L	N_{mol}	Time / ps		
		$N_{trn} = 50$, $L(\Omega) = 0.00005$ Ha, $p_h^A = 2$		$N_{trn} = 500$, $L(\Omega) = 0.00005$ Ha, $p_h^A = 2$
		Flexible	Rigid	Flexible
1	25	1000	1000	1000
	50	1000	1000	1000
	100	1000	1000	1000
	216	100	100	100
2	25	1000	1000	1000
	50	1000	1000	1000
	100	150	150	150
3	25	1000	1000	1000
	50	350	350	
	100	50		

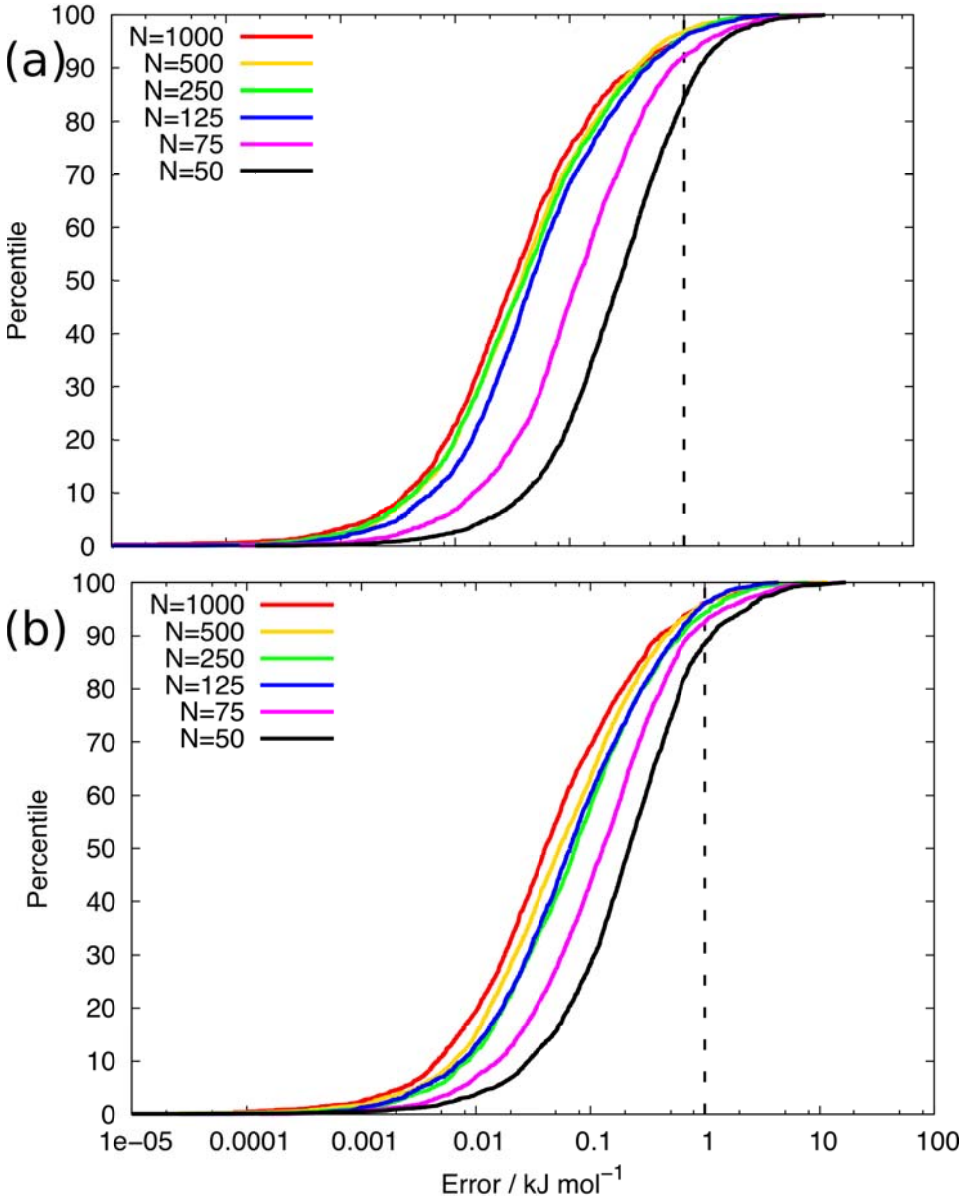

Figure S1. S-Curves showing the prediction error of the sum of atomic E_{IQA}^{A} energies for models constructed with varying N_{trn}, and with p_{h}^{A} fixed at 2 for (a) $L(\Omega)$ = 0.00005 and (b) $L(\Omega)$ = 0.0001 Ha.

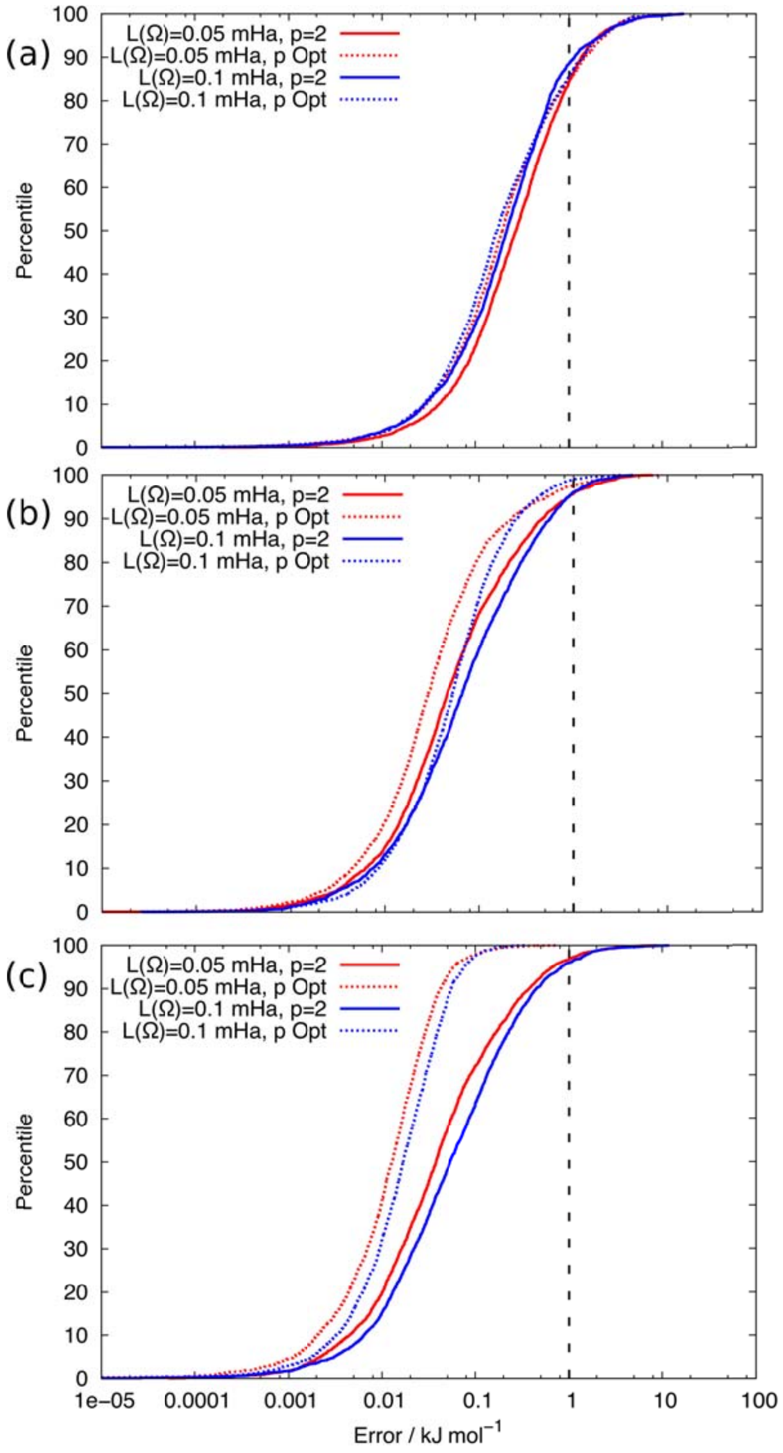

Figure S2. S-Curves showing the prediction error of the sum of atomic E_{IQA}^{A} energies for models constructed with varying $L(\Omega)$ and with p_h^A fixed at 2 or optimized and with N_{trn} equal to (a) 50, (b) 125, and (c) 500.

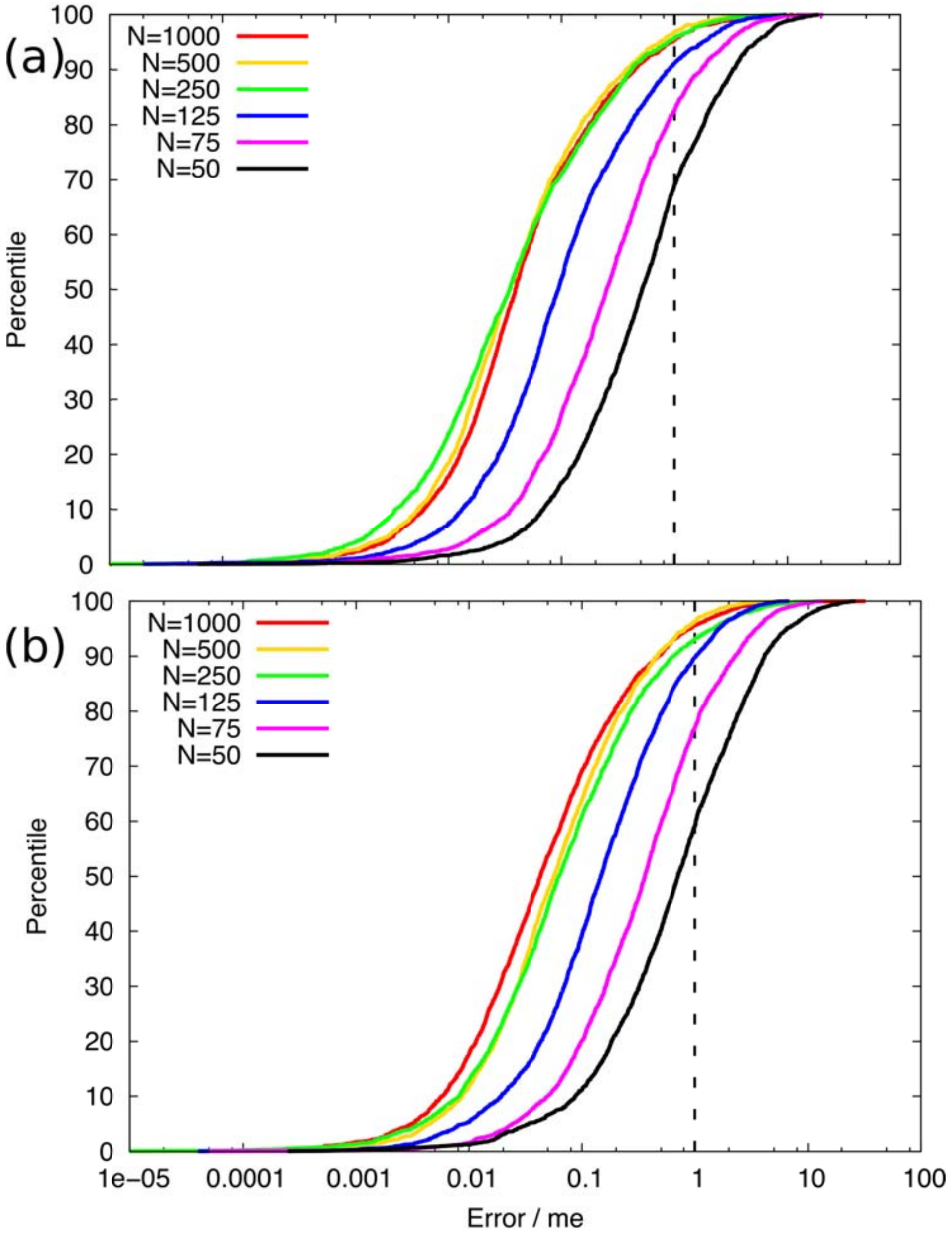

Figure S3. S-Curves showing the prediction error of the sum of atomic charges for models constructed with varying N_{trn}, and with p_h^A fixed at 2 for (a) $L(\boldsymbol{\Omega})$ = 0.00005 and (b) $L(\boldsymbol{\Omega})$ = 0.0001 Ha.

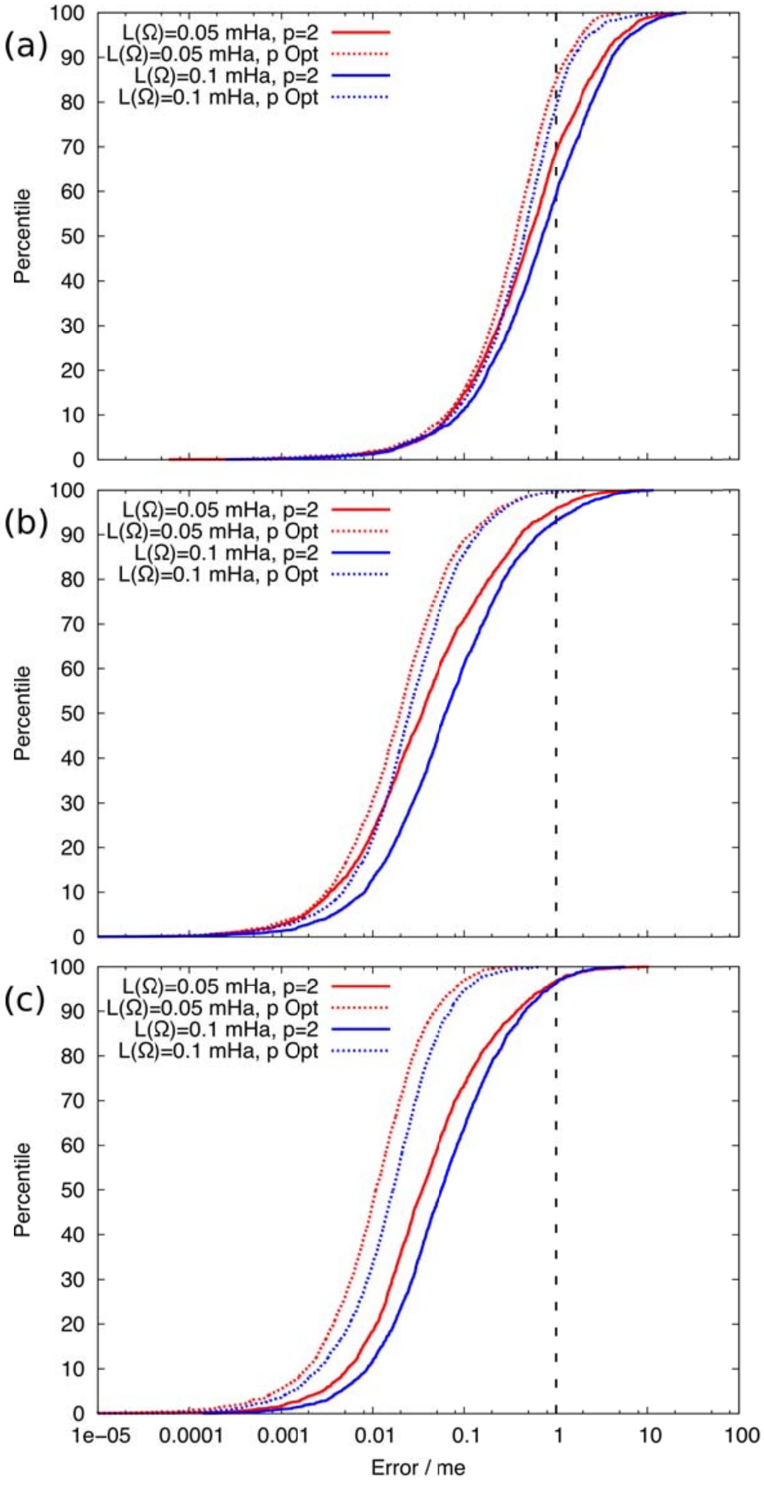

Figure S4. S-Curves showing the prediction error of the sum of atomic charges for models constructed with varying $L(\boldsymbol{\Omega})$ and with p_h^A fixed at 2 or optimized and with N_{trn} equal to (a) 50, (b) 125, and (c) 500.

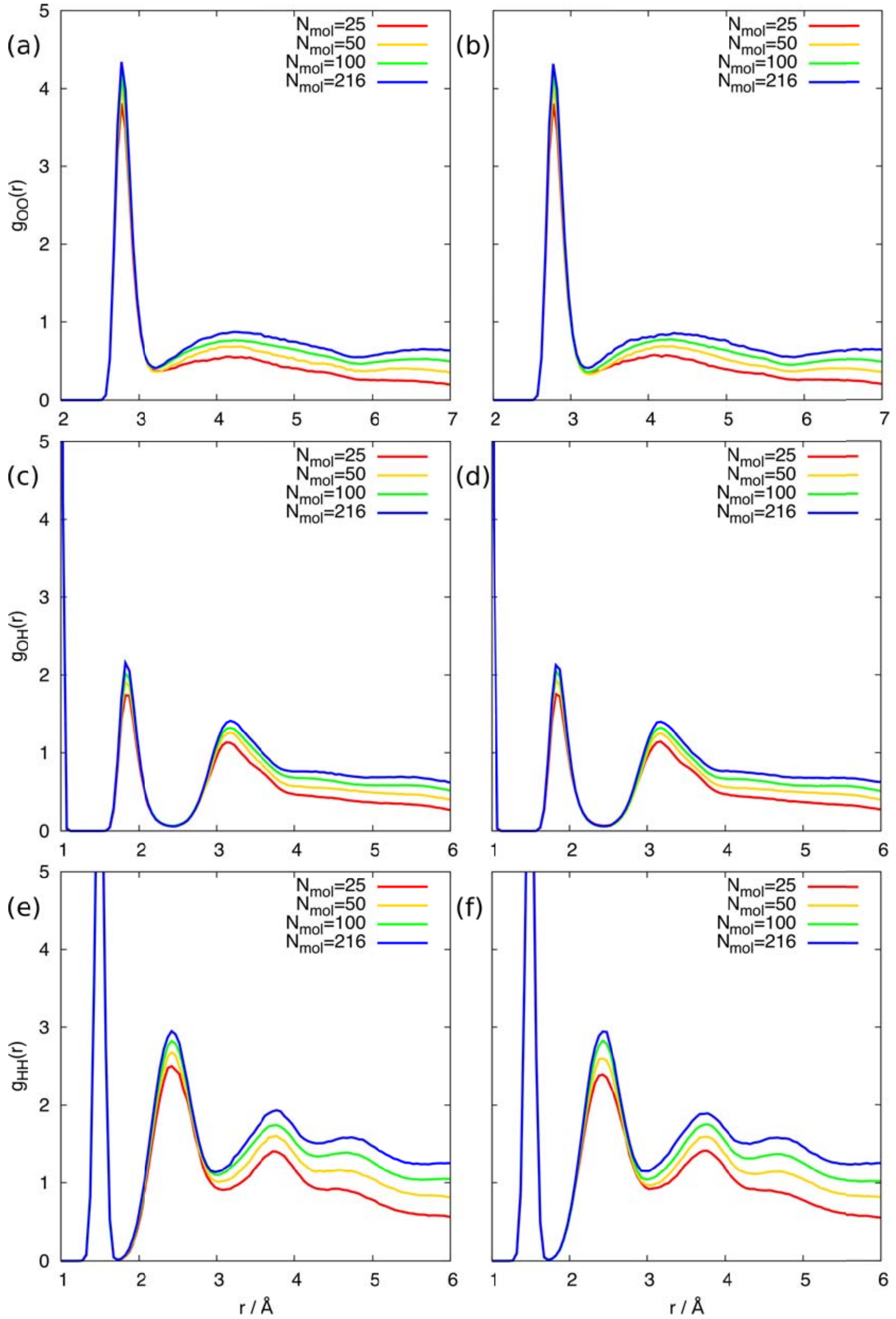

Figure S5. $g(r)$ profiles for L=1 systems with varying N_{mol}: (a) and (b) $g_{\mathrm{OO}}(r)$, (c) and (d) $g_{\mathrm{OH}}(r)$, (e) and (f) $g_{\mathrm{HH}}(r)$. Profiles (a), (c) and (e) are for the model with N_{trn}=50, Profiles (b), (d) and (f) are for the model with N_{trn}=500.

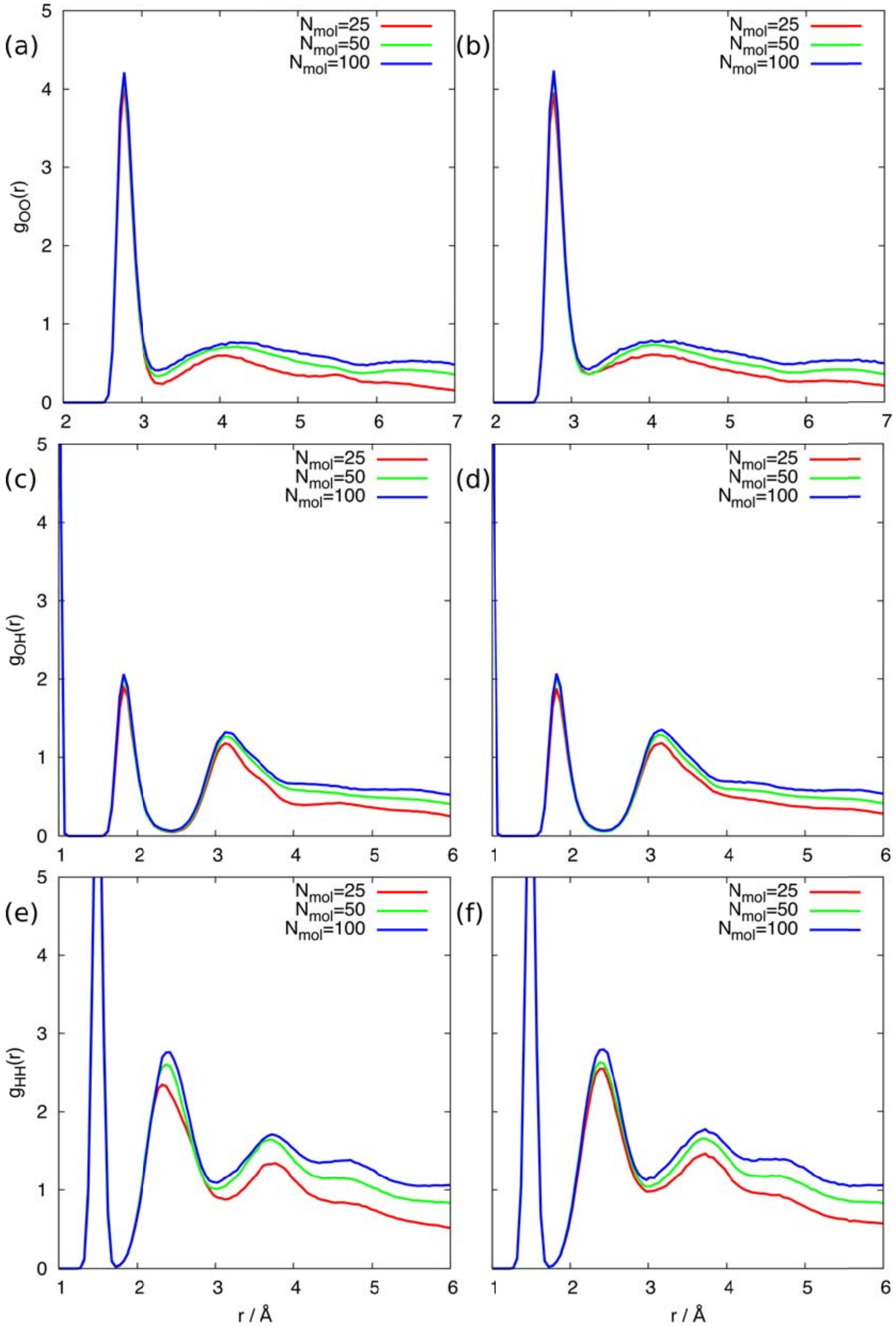

Figure S6. $g(r)$ profiles for L=2 systems with varying N_{mol}: (a) and (b) $g_{\text{OO}}(r)$, (c) and (d) $g_{\text{OH}}(r)$, (e) and (f) $g_{\text{HH}}(r)$. Profiles (a), (c) and (e) are for the model with N_{trn}=50, Profiles (b, (d) and (f) are for the model with N_{trn}=500.

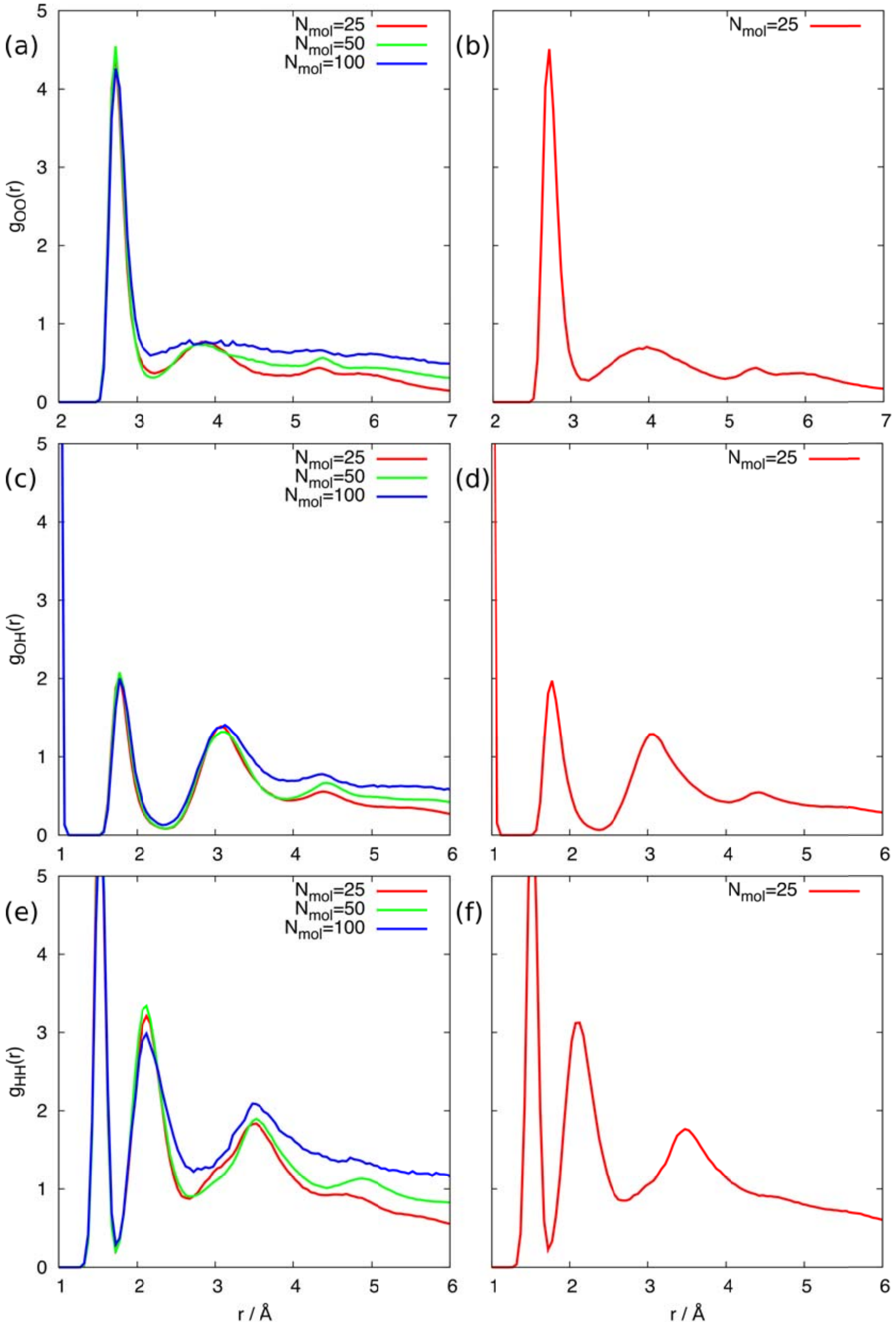

Figure S7. $g(r)$ profiles for L=3 systems with varying N_{mol}: (a) and (b) $g_{\mathrm{OO}}(r)$, (c) and (d) $g_{\mathrm{OH}}(r)$, (e) and (f) $g_{\mathrm{HH}}(r)$. Profiles (a), (c) and (e) are for the model with N_{trn}=50. Profiles (b), (d) and (f) are for the model with N_{trn}=500.

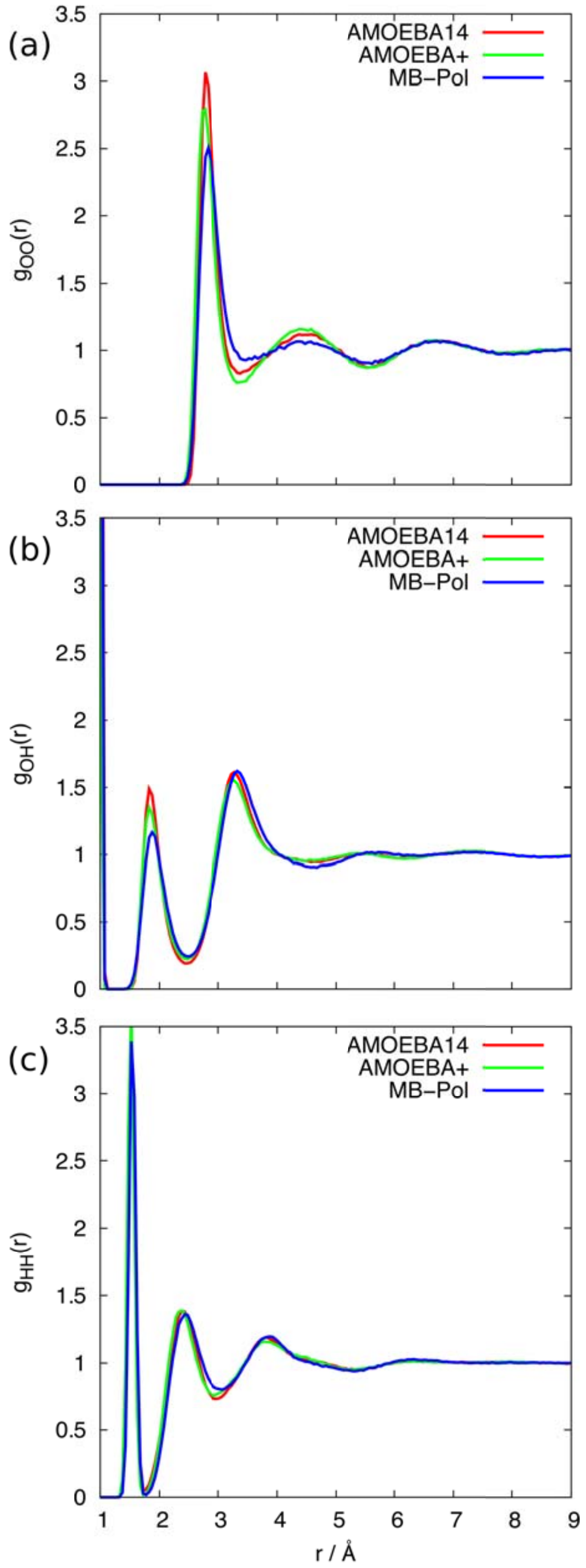

Figure S8. $g(r)$ profiles for bulk water with the AMOEBA14, AMOEBA+ and MB-pol water models: (a) and (b) $g_{OO}(r)$, (c) and (d) $g_{OH}(r)$, (e) and (f) $g_{HH}(r)$.

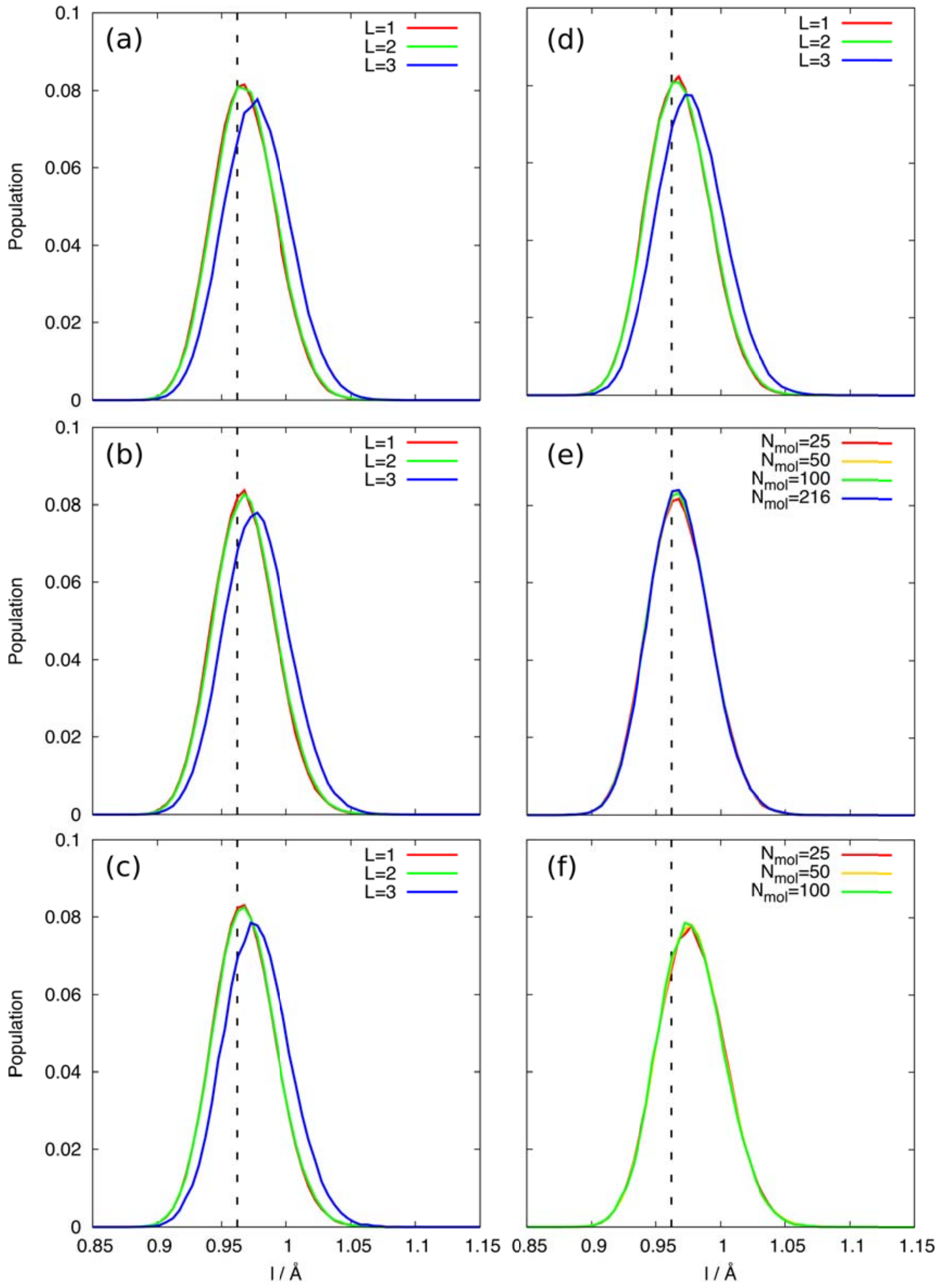

Figure S9. Probability distribution profiles of the O-H bond lengths: FFLUX models with N_{trn}=50 for systems with N_{mol} of (a) 25, (b) 50 and (c) 100. (d) FFLUX models with N_{trn}=500 and N_{mol}=25. (e) FFLUX models with L=1 and N_{trn}=50. (f) FFLUX models with L=3 and N_{trn}=50. The dashed line indicates the B3LYP/aug-cc-pVTZ equilibrium bond length for a single water molecule.

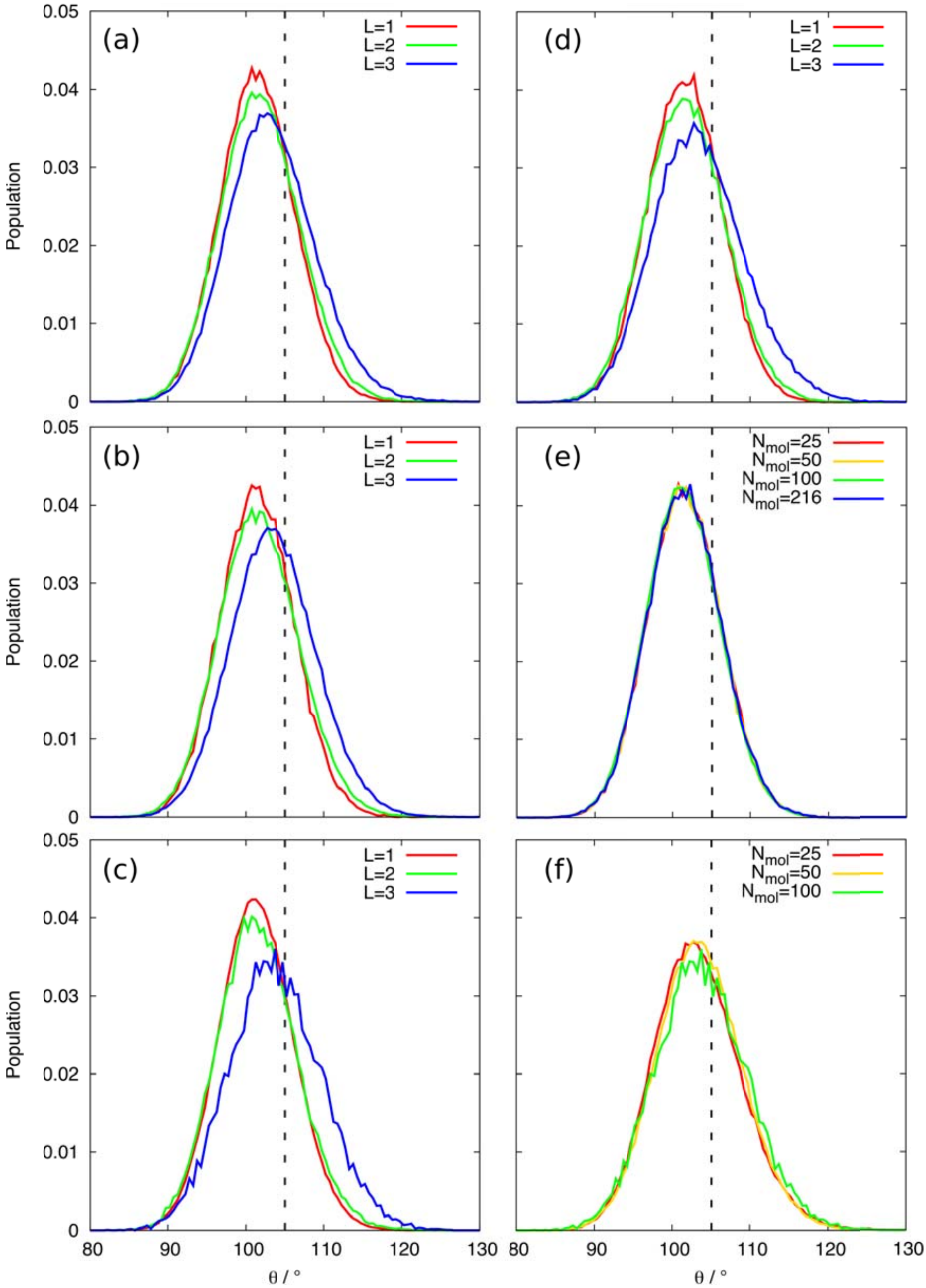

Figure S10. Probability distribution profiles of the HOH bond angle: FFLUX models with N_{trn}=50 for systems with N_{mol} of (a) 25, (b) 50 and (c) 100. (d) FFLUX models with N_{trn}=500 and N_{mol}=25. (e) FFLUX models with L=1 and N_{trn}=50. (f) FFLUX models with L=3 and N_{trn}=50. The dashed line indicates the B3LYP/aug-cc-pVTZ equilibrium bond angle for a single water molecule.

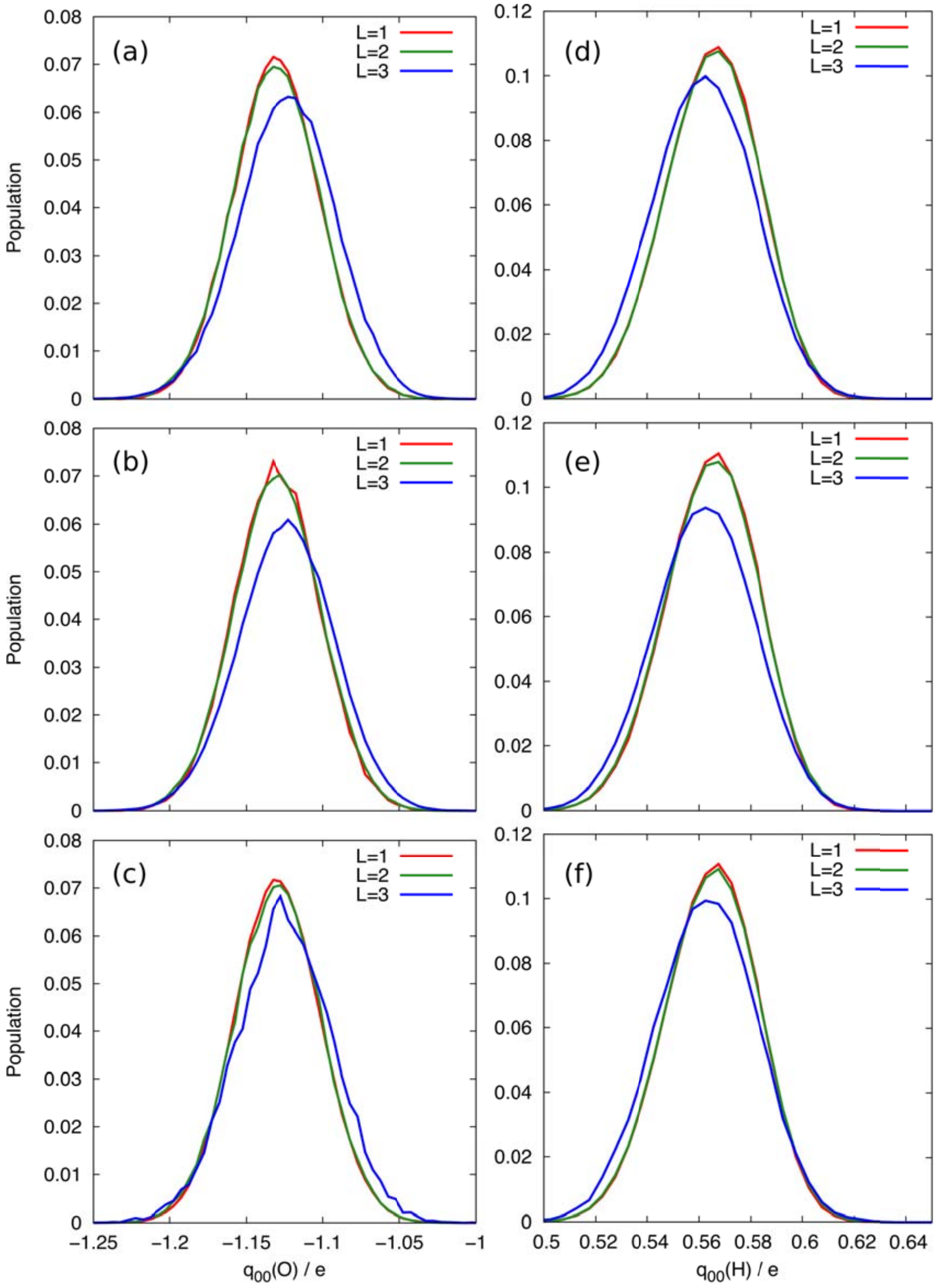

Figure S11. Probability distribution profiles of the atomic charges on the oxygen ((a), (b) and (c)) and hydrogen ((d), (e) and (f)) atoms from the simulations of the FFLUX modes. (a) and (d) N_{mol}=25, (b) and (e) N_{mol}=50 and (c) and (f) N_{mol}=100.

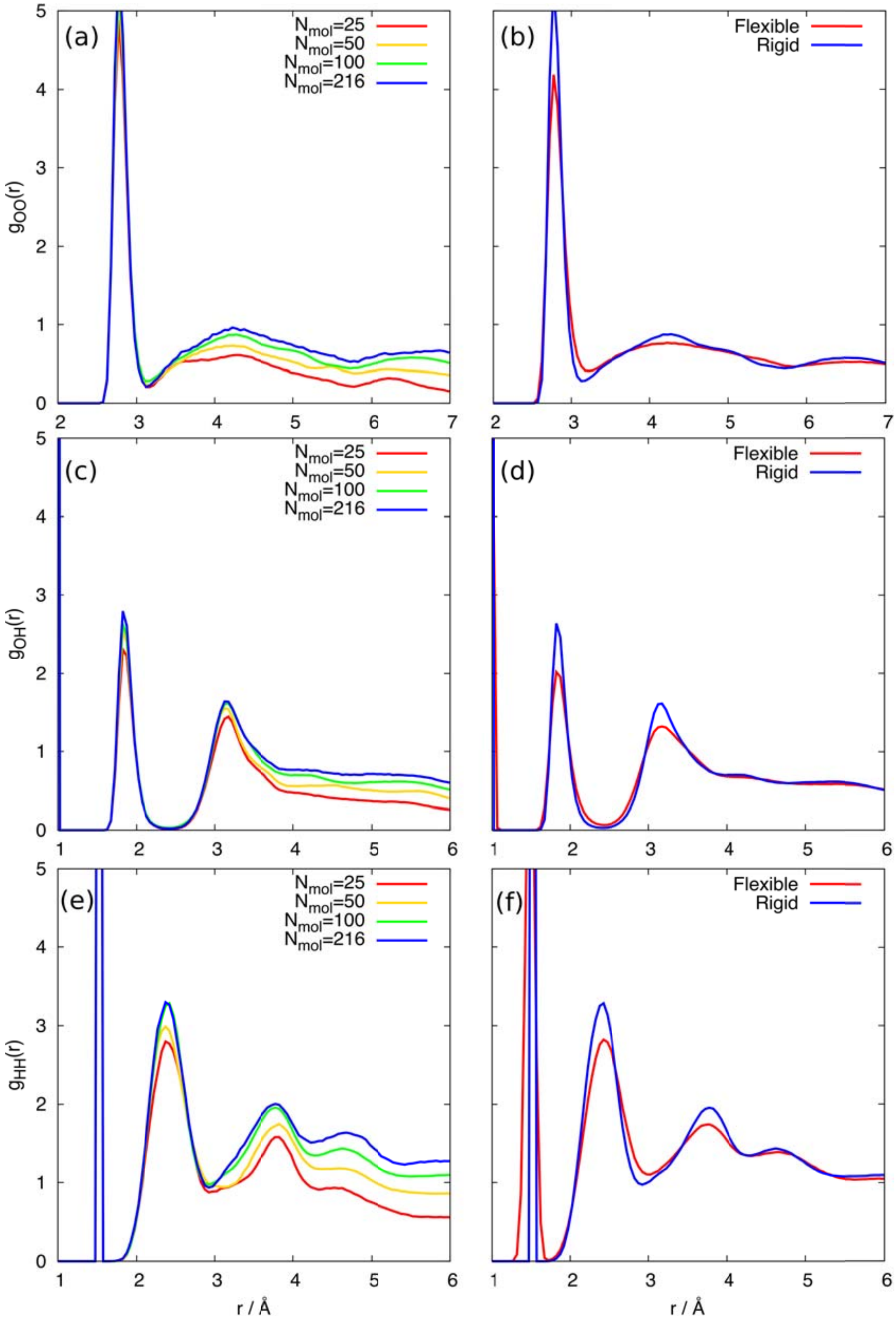

Figure S12. $g(r)$ profiles for L=1 FFLUX models: (a, c, e) rigid models with varying N_{mol}; (b, c, e) rigid and flexible models with N_{mol} = 100. (a) and (b) $g_{\mathrm{OO}}(r)$, (c) and (d) $g_{\mathrm{OH}}(r)$, (e) and (f) $g_{\mathrm{HH}}(r)$.

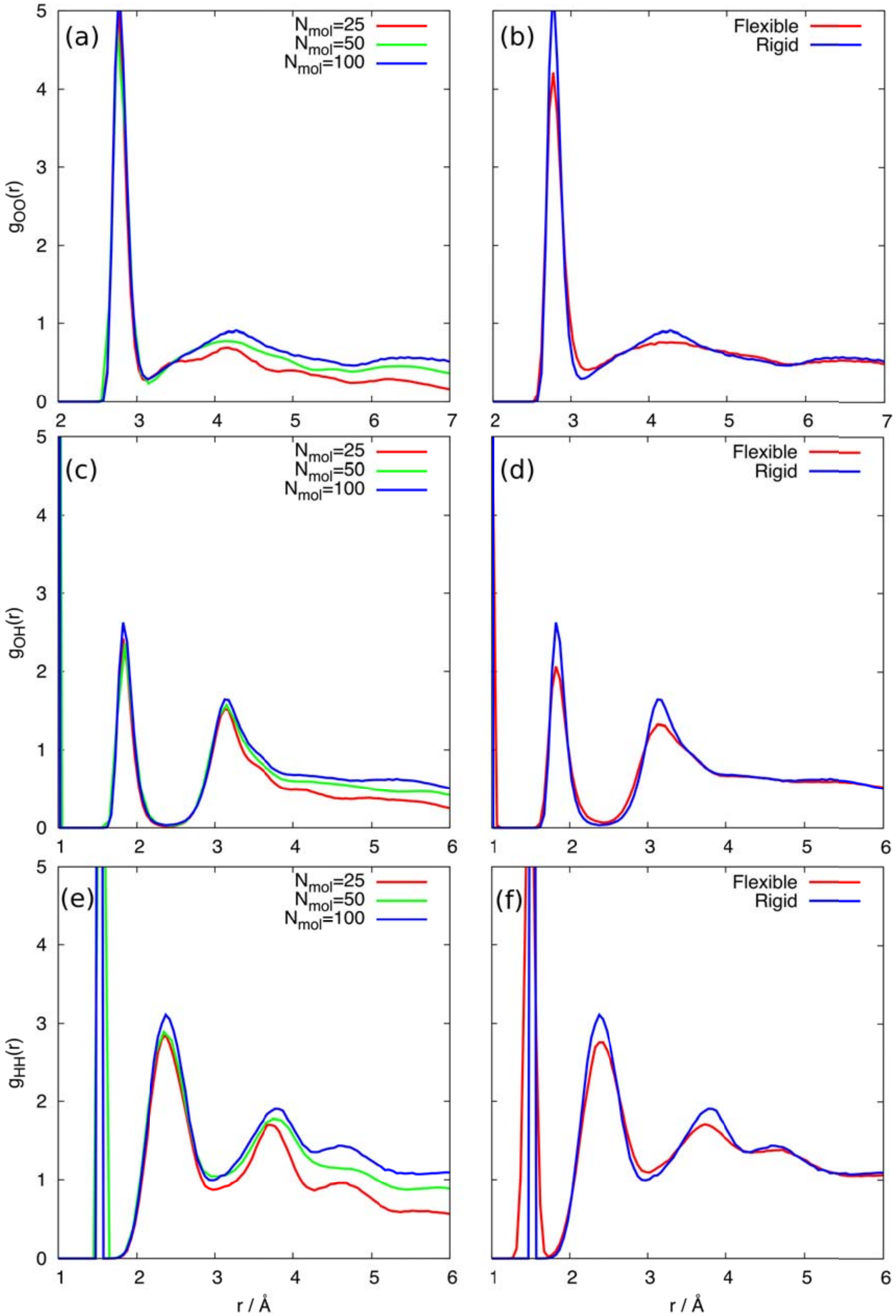

Figure S13. $g(r)$ profiles for L=2 FFLUX models: (a, c, e) rigid models with varying N_{mol}; (b, d, e) rigid and flexible models with N_{mol} = 100. (a) and (b) $g_{\mathrm{OO}}(r)$, (c) and (d) $g_{\mathrm{OH}}(r)$, (e) and (f) $g_{\mathrm{HH}}(r)$.

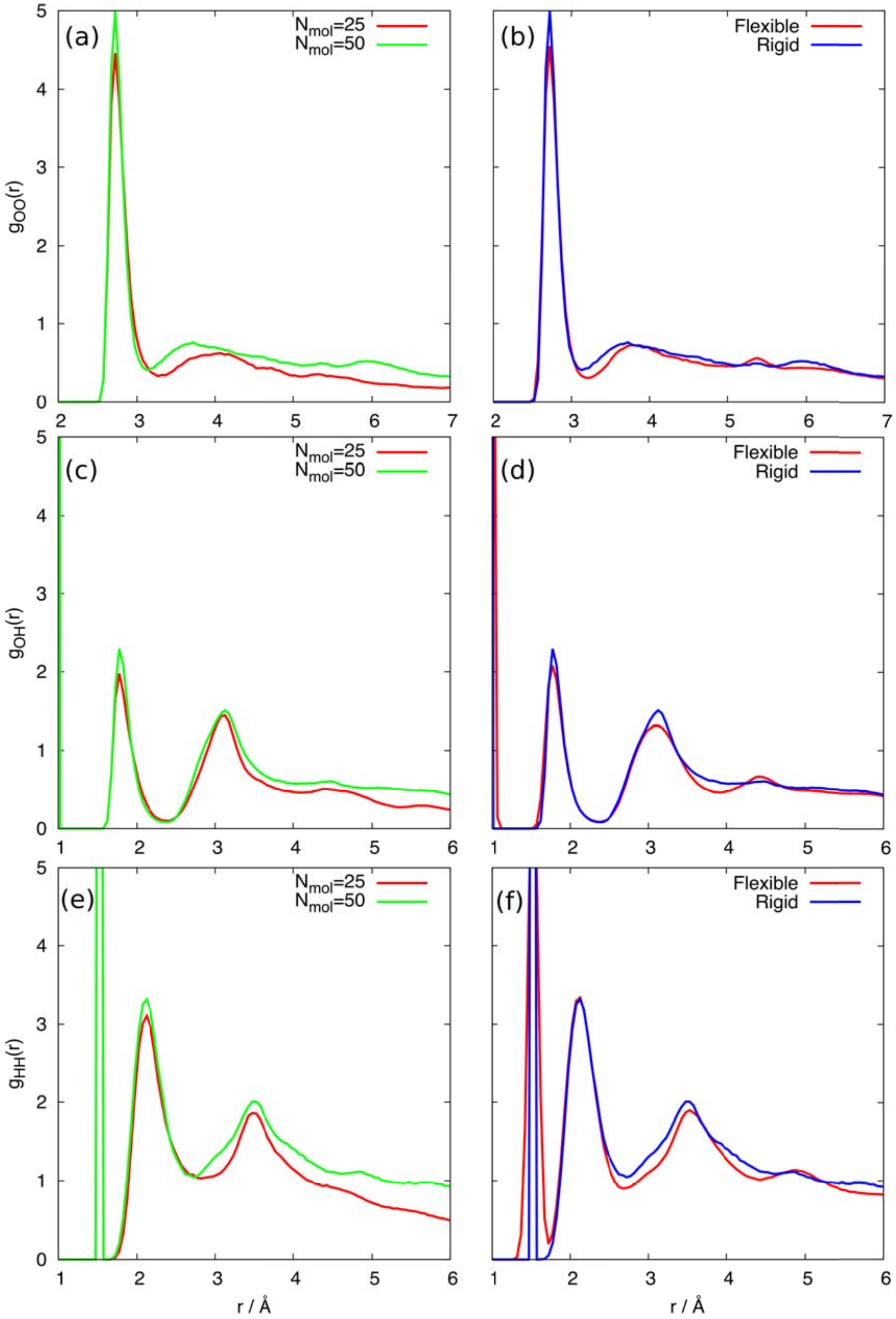

Figure S14. $g(r)$ profiles for L=3 FFLUX models: (a, c, e) rigid models with varying N_{mol}; (b, d, e) rigid and flexible models with N_{mol} = 50. (a) and (b) $g_{\mathrm{OO}}(r)$, (c) and (d) $g_{\mathrm{OH}}(r)$, (e) and (f) $g_{\mathrm{HH}}(r)$.
